# Highly efficient removal of phospholipids in the degumming of crude rapeseed oil using APTES-modified silica adsorbent

**DOI:** 10.1016/j.fochx.2026.104407

**Published:** 2026-09-03

**Authors:** Meihong Wu, Ting Mo, Maoting Chen, Shuang Wang, Qiao Dai, Tongwei Guan, Changsheng Liu, Loubna Arab, Xuemei Liao, Wenlin Li

**Affiliations:** a School of Food and Biological Engineering, Xihua University, 610039 Chengdu, China; b Oil Crops and Lipids Process Technology National & Local Joint Engineering Laboratory, Key Laboratory of Oilseeds Processing, Ministry of Agriculture and Rural Affairs, Oil Crops Research Institute, Chinese Academy of Agricultural Sciences, Wuhan 430062, China; c Yibin Xihua University Research Institute, Yibin 644001, China; d Laboratory of Membrane Processes and Techniques of Separation and Recovery (LPMTSR), Faculty of Technology, Abderrahmane Mira University of Bejaia, 06000, Algeria.

**Keywords:** Removal of phospholipids, Degumming, Oil refining, Hot-pressed rapeseed oil, Silica adsorbent

## Abstract

A new adsorbent, 3-aminopropyltriethoxysilane (APTES) modified silica achieves 98.7% of phospholipid removal and 92.3% of total phenol retention for degumming of crude rapeseed oil at 45 °C for 30 min with dosage of 1% wt. The key indicators of oil quality for APTES-SiO_2_ degummed sample improved significantly, including peroxide value, acid value, color, and clarity. Moreover, *E*-nose and two-dimensional gas chromatography–mass spectrometry analysis results revealed that APTES-SiO_2_ degummed oil had little change of the flavor. FTIR results suggested no trace of APTES molecule residence in the degummed oil, which guaranteed the oil safety. Combined with the correlations of zeta potential, XPS results, and degumming performance, the adsorption mechanism revealed that the hydrophobic surface of APTES-SiO_2_ adsorbent worked synergistically with protonated amino groups to promote dephosphorization rate. Our findings provided high interest for rational design and preparation of highly dispersed and efficient adsorbent for degumming of crude oil.

## Graphical abstract

**Figure:**
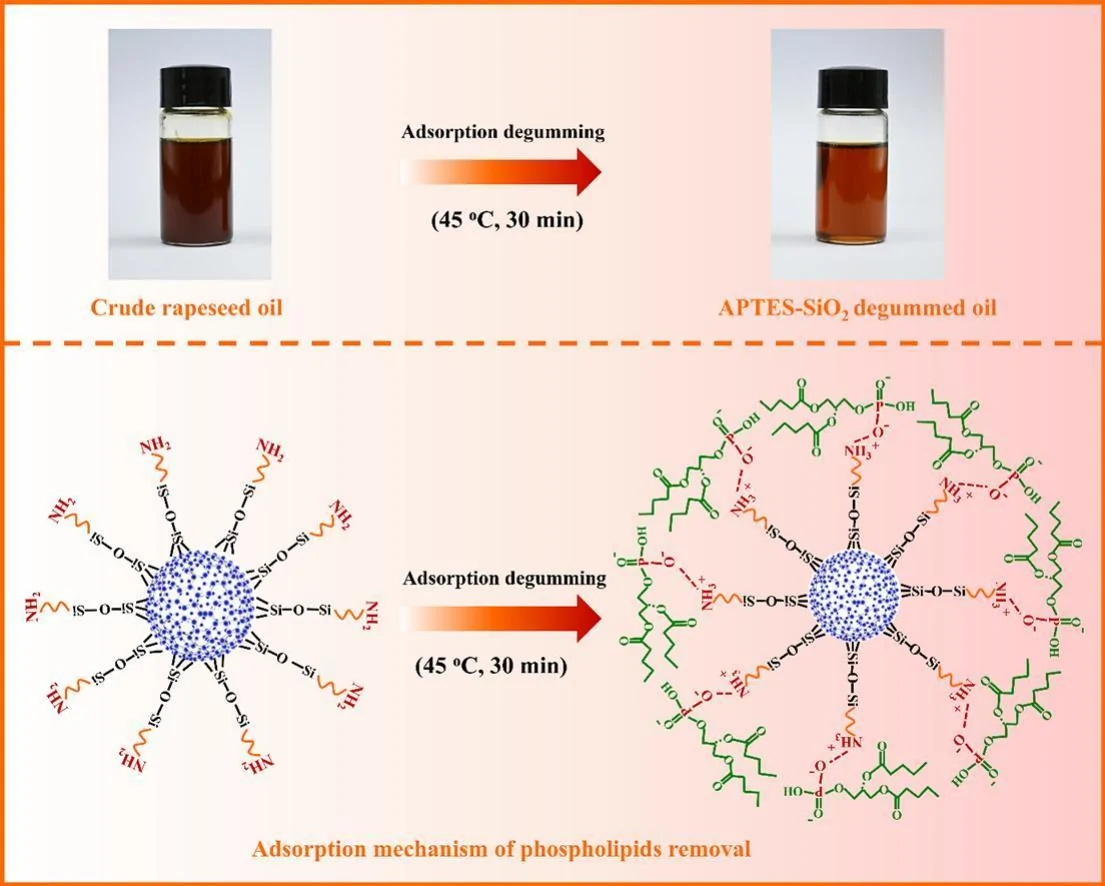

## 1 Introduction

Rapeseed oil is recognized as one of the world's three major edible oils due to its rich nutritional composition and distinctive flavor profile. Refined rapeseed oil is typically produced from crude rapeseed oil through five subsequent processes: degumming, deacidification, dewaxing, bleaching, and deodorization (Lv et al., 2025). Among these, degumming aims to remove phospholipids, as their presence poses several potential risks: (1) phospholipids compromise the oil's high-temperature stability, making it prone to oxidation, darkening, and carbonization, and can cause smoking and foaming during cooking; (2) they accelerate oxidation and discoloration during storage, shortening the oil's shelf life; (3) inadequate phospholipid removal during degumming can adversely affect subsequent refining steps (Cheng et al., 2025; Liang et al., 2023). Therefore, the effective removal of phospholipids from crude rapeseed oil has become a critical issue in oil refining.

Currently, industrial degumming processes mainly rely on water and acid degumming (Feng et al., 2026; Lv et al., 2025). These traditional methods generate chemical wastewater, require high temperatures (>75 °C), and consume considerable energy, which are not conducive to environmental protection or sustainable development (Feng et al., 2026; Wang, Sun, Yao and Chu, 2021). The recent film degumming can replace the old energy-intensive methods, but the organic film has low stability, low penetration flux, and high oper-ating expenses (Pan et al., 2025). Although enzymatic degumming has been regarded as a greener alternative for phospholipid removal, achieving a dephosphorization rate of over 90%, this approach remains cost-prohibitive for industrial application (L. Zhang et al., 2022). Adsorption degumming technology has attracted increasing attention due to its high efficiency, low energy consumption, and environmental friendliness. Commonly used adsorbents for this purpose include sepiolite, clay, activated carbon, and silicon dioxide (SiO_2_). Among these, the application of SiO_2_ in oil degumming has drawn considerable interest. Our previous results showed that SiO_2_ degumming achieved an 85% dephosphorization rate and a high retention rate of total phenols (up to 96%) (Yao et al., 2020). In another study, after water degumming of okra seed oil followed by SiO_2_ degumming, the maximum phospholipid removal rate was only 43%, with only 62% of phenolic compounds retained. Meanwhile, the peroxide value increased (Xiong et al., 2022). Zhang et al. investigated a silica hydrogel for degumming hot-pressed aromatic rapeseed oil (W. Zhang et al., 2024). They found that under conditions of 45 °C for 15 min with addition of 1.1% wt silica hydrogel, the phospholipid removal rate reached 97%; however, the micronutrient retention rate was only 85%, and the volatile flavor characteristic components of the refined oil were weakened.

The aforementioned SiO_2_ contains a large number of silanol (Si-OH) groups on its surface, in addition to its porous properties, which are believed to effectively adsorb both hydratable and non-hydratable phospholipids (NHPs) without the need for water or acid reagents (Alhikami et al., 2026). However, the abundant hydrophilic hydroxyl groups readily cause SiO_2_ particles to aggregate in hydrophobic systems, i.e., in the oil phase (Fu et al., 2023), resulting in poor mass transfer. To improve adsorption performance, surface modification of SiO_2_ has been developed to enhance the number and activity of adsorption sites. For example, SiO_2_ modified with grafted amino-polycarboxylic acid ligands can convert NHPs into hydratable phospholipids (HPs), which are subsequently removed, achieving a 63% removal rate in soybean oil via hydration degumming (Pan et al., 2025). Another modification of SiO_2_ involves usage of NaOH, which introduces Na^+^ ions capable of inter-acting with oxygen atoms, thereby enhancing surface reactivity (Rimsza et al., 2018; Y. Wang et al., 2024). However, due to the weak interaction between the modifier and the adsorbent, leaching of the modifier into the refined oil cannot be ruled out, potentially leading to food safety concerns. It has been reported that amine-functional groups modified silica via 3-aminopropyltriethoxysilane (APTES) is effective in crude oil purification applications (Alhikami et al., 2026). For example, APTES-impregnated mesoporous hollow silica showed much higher adsorption capacity of free fatty acids (FFAs) than that of silica microspheres (Y. Wang et al., 2024).

To construct an ideal silica-based adsorbent for phospholipid removal, we selected the silane coupling agent APTES to graft onto the SiO_2_ surface via the formation of Si-O-Si covalent bonds, thereby increasing the interaction between the modifier and the adsorbent and creating favorable adsorption sites. Additionally, the APTES molecule contains a hydrophobic hydrocarbon chain (e.g., propyl chain) bearing an amino group, which is expected to enhance the dispersion of the APTES-SiO_2_ adsorbent in rapeseed oil and thus promote the mass transfer of non-hydratable phospholipids toward the adsorbent surface. As a result, under the optimized adsorption conditions (45 °C, 30 min, 1 wt% dosage), APTES-modified silica achieved a phospholipid content of only 45 mg/kg after degumming, compared to 1032.8 mg/kg for its unmodified silica counterpart. The key quality indicators of the APTES-SiO_2_ degummed oil improved significantly, while the flavor was well retained. Extensive characterizations, including scanning electron microscopy-energy dispersive spectroscopy (SEM-EDS), contact angle measurements, zeta potential, and X-ray photoelectron spectroscopy (XPS), were applied to establish the relationship between the physicochemical properties of the adsorbent and its adsorption performance, as well as to elucidate the adsorption mechanism.

## 2 Materials and methods

### 2.1 Materials and reagents

Crude rapeseed oil was sourced from Chengdu Fuyoufang Grain and Oil Co., Ltd. Crude okra seed oil was obtained from the Institute of Agricultural Processing, Sichuan Academy of Agricultural Sciences. Edible-grade silica dioxide (purity≥99.0%) was procured from Qingdao BRT Silica Technology Co., Ltd. (Qingdao, China). The APTES (3-aminopropyltriethylsilane, 99%) was procured from Shanghai Aladdin Biochemical Technology Co., Ltd. Isopropyl alcohol (purity≥99.7%) was sourced from Tianjin Fuyu Fine Chemical Co., Ltd. The acetone (purity≥99.5%) was obtained from Chengdu Jinshan Chemical Reagent Co., Ltd. The gallic acid standard was procured from Shanghai Maclin Biochemical Technology Co., Ltd. The other reagents were acquired from Chengdu Colon Chemical Co., Ltd.

### 2.2 Preparation of APTES modified silica

The weighed silica powder of 2 g was dispersed under stirring in 50 mL isopropyl alcohol in a round-bottom flask. Subsequently, 2 mL of APTES were introduced into the dispersion medium and maintained under reflux conditions at 85 °C for 15 h. After the reaction, the solution and solid powder were separated by filtration. The obtained solid paste was washed with isopropyl alcohol, followed being dried at 90 °C in the oven overnight. The obtained sample was named APTES-SiO_2_.

### 2.3 Degumming process

A total of 100 mL of crude rapeseed oil was mixed with different mass of adsorbent varied as 0.5 g, 1 g, 1.5 g, 2 g, 2.5 g. The degumming process was carried out at 45 °C for 20, 30, 40, 50 min. Following adsorption, the mixture was centrifuged at 10,000 rpm for 20 min, and the supernatant was collected as the degummed oil. The degummed oil sample was stored in a refrigerator at 4 °C for 15 h prior to subsequent analyses.

### 2.4 Adsorbent characterization

The sample was immobilized on conductive adhesive, and the surface morphology of SiO_2_ and APTES-SiO_2_ adsorbents before and after modification was obtained by scanning electron microscopy (VEGA3 SBH, TESCAN, Czech Republic) coupled with energy dispersive spectroscopy (EDS) spectrometer, using an accelerating voltage of 30 kV.

The pore structure parameters of the adsorbent, including the Brunauer-Emmett-Teller (BET) specific surface area, pore volume, and average pore diameter, were determined via N₂ adsorption-desorption measurements performed at −196 °C using a JW-BK122W physical adsorption analyzer. Prior to analysis, all samples were degassed at 100 °C for 3 h to remove adsorbed impurities and moisture.

The contact angle (CA) at the three-phase interface of SiO_2_ and APTES-SiO_2_ was determined using an optical contact angle meter and interfacial tension analyzer (SL200KS, KINO, USA). Press approximately 150 mg of the sample powder into a 1 mm thick pellet using a tablet press. Immerse the thin section in rapeseed oil for 15 s, then remove it. Subsequently, use a high-precision syringe to dispense 5 μL of ultrapure water onto the surface of the thin section. Droplet images were subsequently captured with a camera, and the contact angles were calculated by analyzing the images with the instrument's built-in software. All measurements were performed at 25  °C, with each sample measured at least three times (Tao et al., 2024).

The chemical structure of the SiO_2_, APTES-SiO_2_ adsorbent before and after adsorption, and rapeseed oil were characterized by Fourier transform infrared spectroscopy (FTIR, PerkinElmer Inc., USA), carrying on a Spectrum Two Fourier transform infrared spectrometer (Spectrum Two, PerkinElmer Inc., USA) in the 4000–400 cm^−1^ wavenumber range using the KBr pellet technique at room temperature.

The surface charge of the adsorbent was evaluated through zeta potential measurements using a Zeta potential analyzer (Nano-ZS, Malvern Instruments Ltd., Worcestershire, UK). Disperse and dilute the following four samples approximately 1000 times using neutral phosphate buffer solution: APTES-SiO_2_, APTES-SiO_2_ after adsorbing crude oil, APTES-SiO_2_ after adsorbing crude oil and oleic acid mixed oil samples, and APTES-SiO_2_ after adsorbing oleic acid. All the samples were diluted with n-hexane under ultrasonic treatment for 3 min to remove the bubbles. A measured volume of the sample solution was introduced into the sample cell for testing at 25 °C. The average of three consecutive measurements was recorded as the final result.

The chemical state of nitrogen in APTES-SiO₂ was characterized using X-ray photoelectron spectroscopy (XPS) with a Thermo Scientific K-Alpha spectrometer (Thermo Fisher Scientific, America) equipped with an Al-Kα X-ray source (1486.6 eV), 150 W power, and a 400 μm spot size. The C 1 s photoelectron peak was employed as an internal reference for spectral calibration, with a binding energy of 284.8 eV assigned to adventitious carbon (Wang et al., 2021).

### 2.5 Physicochemical properties of rapeseed oil

The determination of phospholipid content was based on methods by AOCS official method Ja 4–46 (AOCS 2003), with certain modifications (Garba et al., 2020). In order to evaluate the reliability of this method, the phospholipid content determined by the molybdenum blue colorimetric method in accordance with the AOCS official method Ca 12–55 (AOCS, 2009) was also compared. As shown in Table S1, the phospholipid content was determined to be 3260 mg/kg by the acetone gravimetric method and 3391 mg/kg by the molybdenum blue colorimetric method, respectively, indicating that the gravimetric method employed in this study demonstrates a high degree of reliability.

The acid value was determined via titration with potassium hydroxide, following the AOCS official method Cd 3d-63 (AOCS, 1997). The peroxide value of rapeseed oil was determined using the acetic acid-chloroform method in accordance with AOCS official method Cd 8–53 (AOCS, 1997). The total phenolic content was determined spectrophotometrically with the Folin-Ciocalteau reagent using an ultraviolet spectrophotometer (UV-1900, Shanghai Yi electric Analytical Instrument Co., LTD, China) (Grzelczyk et al., 2022). The color of rapeseed oil was evaluated using an automatic colorimeter (DC-P3, Beijing Xingguang Colorimeter Instrument Co., Ltd., China). The color characteristics of the oils were further evaluated based on the measured values of the CIE L*, a*, b* color space parameters, where L* represents lightness, a* value is employed to denote the red-green, and the b* value is denoted the blue-yellow.

### 2.6 Electronic nose analysis

The electronic nose (*E*-nose) analysis was performed with minor modifications using an E-nose system (PEN3 E-Nose, Airsense Analytics GmbH, Germany). This instrument features 10 independent metal oxide sensors, each corresponding to specific volatile organic compounds: W1C (aromatics), W5S (nitrogen oxides), W3C (ammonia and aromatics), W6S (hydrogen), W5C (olefins and aromatics), W1S (hydrocarbons), W1W (hydrogen sulfide), W2S (alcohols and partial aromatics), W2W (aromatic components and organic sulfides), and W3S (aromatics). For each type of oil sample (crude rapeseed oil, SiO_2_ degummed oil, and APTES-SiO_2_ degummed oil), 15 mL of each sample was placed in a 50 mL headspace vial and equilibrate at room temperature for 30 min. The headspace gases were then introduced into the E-nose system using a carrier gas flow rate of 400 mL/min. Sensor resistance values were recorded at a sampling frequency of one data point per second over a 120 s period. For analysis, the average stable signal observed between 63 and 65 s was selected, and all measurements were performed in at least three independent replicates (Yao et al., 2020).

Following the method described by Pan et al. with modifications(Pan et al., 2025), headspace solid-phase microextraction coupled with comprehensive two-dimensional gas chromatography–mass spectrometry (GC × GC–MS, Shimadzu GCMS-QP2020NX) was employed to analyze volatile flavor compounds in rapeseed oil. Five grams of each of three rapeseed oil samples (crude oil, SiO_2_-degummed oil, and APTES-SiO_2_-degummed oil) were placed in 20-mL headspace vials. Five microliters of 2-octanol (50 mg/mL) were added as an internal standard, and the vials were sealed and mixed. After equilibration in a 60 °C water bath for 35 min, headspace solid-phase microextraction was performed for 40 min, followed by thermal desorption at 250 °C at the injection port for 5 min. Each sample was analyzed in triplicate.

### 2.7 Adsorption kinetic experiments

The phospholipids in the oil samples were removed via batch adsorption to investigate the adsorption mechanism of the modified adsorbent. For kinetic studies, 0.75 g of the adsorbent was added into 300 mL of solutions with crude rapeseed oil under stirring for different times at 45 °C. Afterwards, the sample was collected, and the adsorbent was separated via centrifugation at 10,000 rpm for 20 min. The experimental data were fitted and analyzed using the quasi-first-order kinetic model and the quasi-second-order kinetic model, respectively, according to the following equations (Tang et al., 2024):

(1) $$\text{Quasi}-\text{first}-\text{order dynamics formula}:q_{t}=q_{e}\left(1-e^{-k_{1}t}\right)$$

(2) $$\text{Quasi}-\text{second}-\text{order dynamics formula}:\frac{t}{q_{t}}=\frac{t}{q_{e}}+\frac{1}{k_{2}q_{e}^{2}}$$

where q_e_ and q_t_ denote the phospholipid adsorption capacities (mg･kg^−1^) at equilibrium and at time t, respectively. k_1_ (mg･kg^−1^･min^−1^) represents the adsorption rate constant of the pseudo-first-order kinetic model. k_2_ (mg･kg^−1^･min^−1^) denotes the adsorption rate constant in the pseudo-second-order kinetic model.

The adsorption degumming process was optimized. Different dosages (0.5–2.5 g) of APTES-SiO_2_ were added to five beakers containing 100 mL of oil sample respectively. Afterwards, degumming process was performed at 45 °C for different adsorption times (20–60 min), followed by centrifugation of the mixture at 10,000 rpm for 20 min. Finally, using the phospholipid content and total phenolic content of the degummed rapeseed oil as evaluation indicators, the degumming process was optimized through single-factor experiments. The phospholipid removal rate was calculated according to the following equation:

(3) $$\text{phospholipid removal rate}\;\left(\%\right)=\frac{m_{0}-m_{1}}{m_{0}}\times 100$$

where m_0_ denotes the phospholipids content of the crude oil (mg/kg), and m_1_ denotes the phospholipids content of the oil after degumming (mg/kg).

## 3 Results and discussion

### 3.1 Microstructure of SiO_2_ and APTES-SiO_2_

**Fig. 1:**
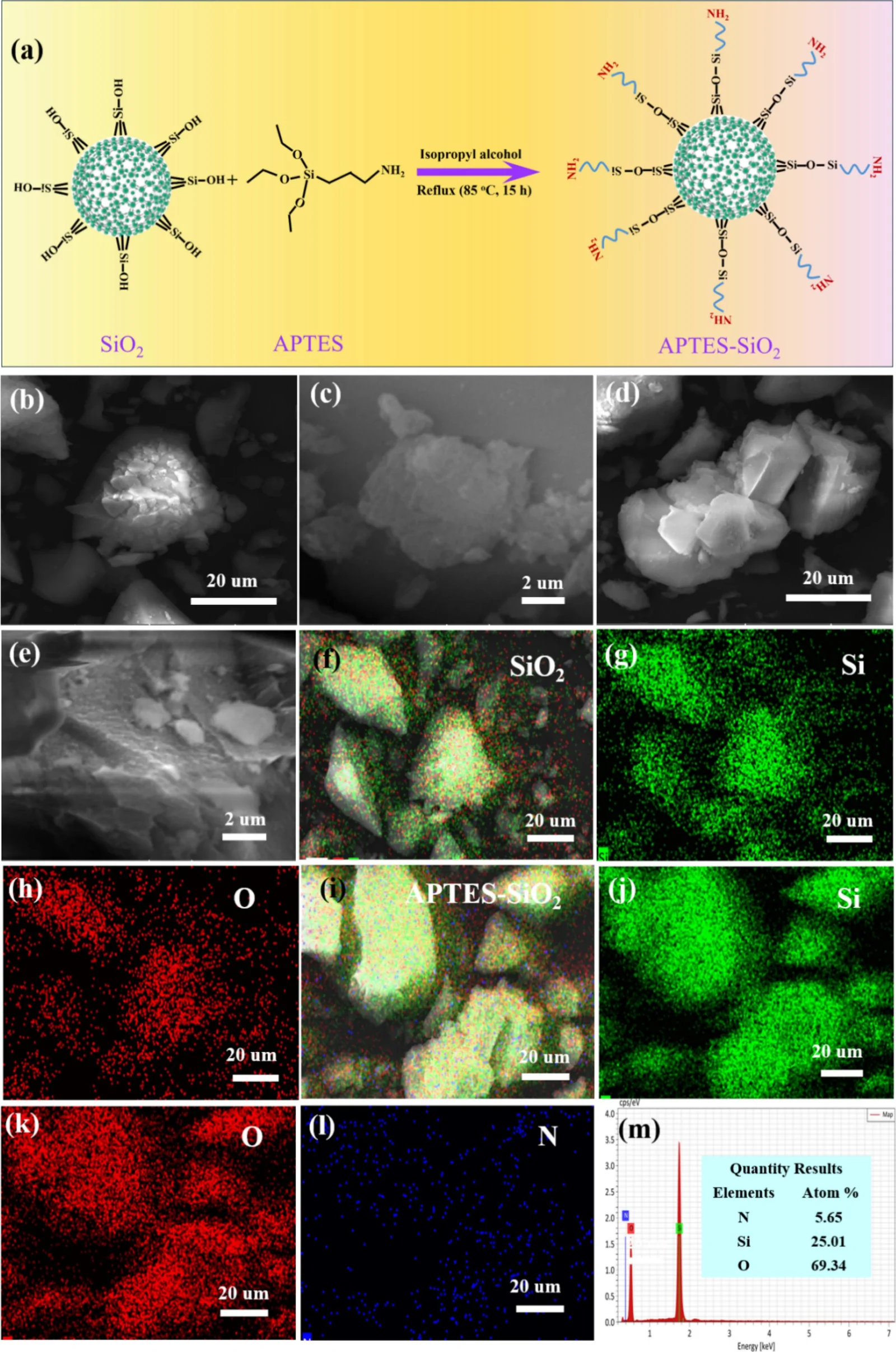
Preparation route schematic illustration of APTES-SiO_2_ (a). SEM images of SiO_2_ (b and c), APTES-SiO_2_ (d and e). EDS analysis of SiO_2_ (f-h) and APTES-SiO_2_ (i-m).

The preparation route for APTES-modified silica is illustrated in Fig. 1a. Under anhydrous conditions, the ethoxy groups of APTES molecules directly react with the Si-OH groups on the silica surface to form Si-O-Si bonds at 85 °C, using isopropyl alcohol as the solvent in a round-bottom flask under reflux. In this synthetic process, APTES molecules are expected to be firmly anchored onto the silica surface via the formation of Si-O-Si covalent bonds.

The microstructure of SiO_2_ and modified SiO_2_ by APTES was characterized by scanning electron microscopy technique (SEM), and images were presented in Fig. 1b-e and Fig. S2. The microstructure of pristine SiO_2_ and APTES-modified SiO_2_ was characterized by scanning electron microscopy (SEM), and the images are presented in Fig. 1b-e and Fig. S2. As can be seen, the pristine SiO_2_ exhibited irregular morphology with various protrusions on the surface and a predominant diameter of approximately 20 μm (Fig. 1b, c and Fig. S3). Compared with the pristine SiO_2_, the APTES-SiO_2_ sample presented a flatter and smoother surface, along with a more uniform particle size distribution. The fractured surfaces were observed to be predominantly covered with micron-sized granules (Fig. 1d, e), similar to results reported in the literature (Lima et al., 2019). To further confirm the grafting of APTES molecules onto SiO_2_, elemental mapping derived from SEM-EDS analysis is shown in Fig. 1f-m. The SiO_2_ sample only displays Si and O elements (Fig. 1g, h). In contrast, the images in Fig. 1i-l show that APTES-modified SiO_2_ contains Si, O, and N elements. The presence of N signals further confirms the attachment of APTES molecules to the SiO_2_ surface. Moreover, nitrogen is homogeneously distributed across the silica surface. The elemental compositions of the two adsorbents are shown in Fig. S4. Compared with unmodified SiO_2_, the Si content of APTES-SiO_2_ decreased from 29.8% to 25.0%, further indicating the introduction of N onto SiO_2_, which is consistent with the results reported by Maria C. Ruiz-Canas et al.(Ruiz-Cañas et al., 2020).

### 3.2 Textural properties and FTIR results of SiO_2_ and APTES-SiO_2_

**Fig. 2:**
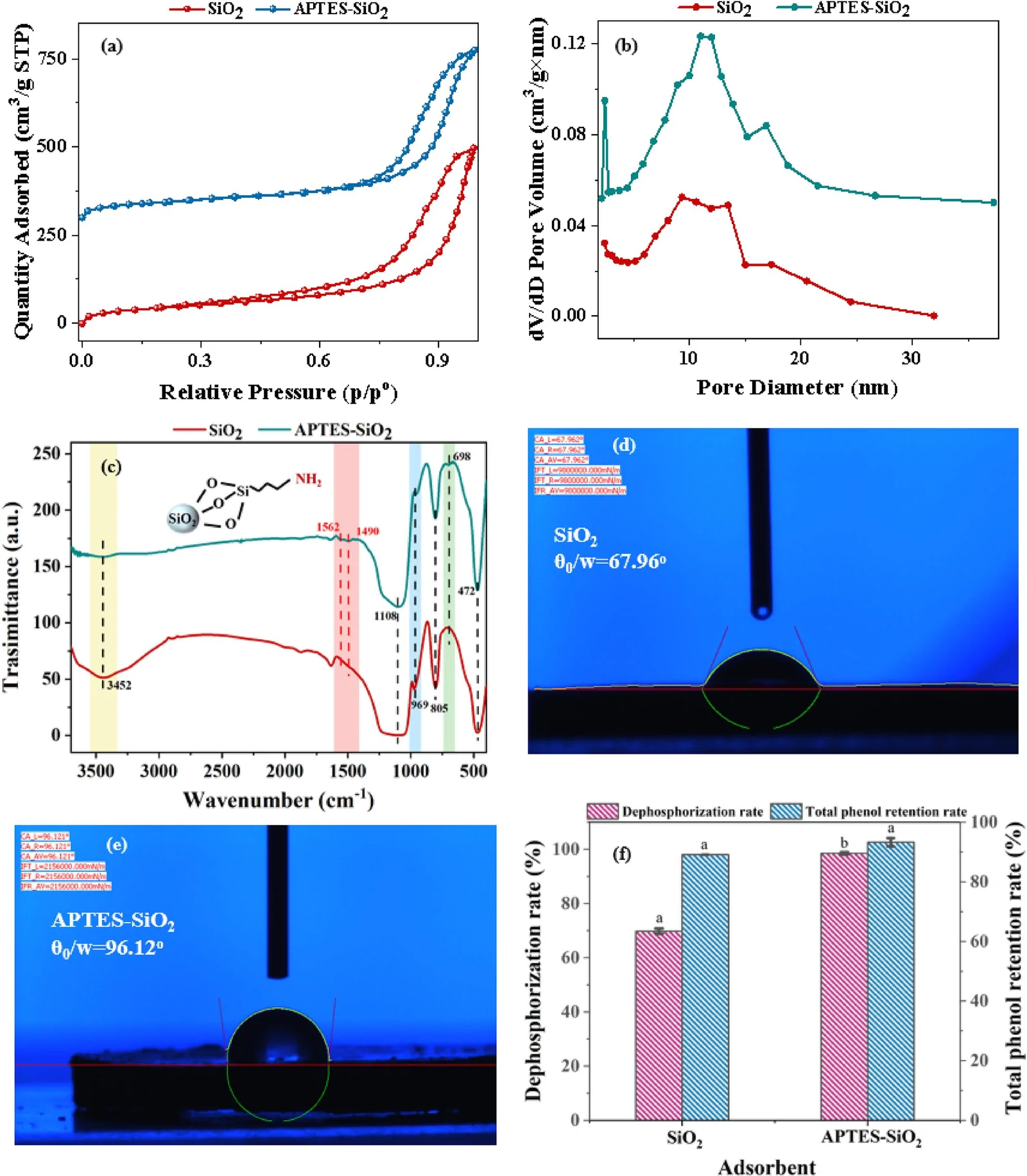
N_2_ adsorption and desorption isotherm of the adsorbents (a), pore size distribution of the adsorbents (b), contact angle measurements of the SiO_2_ (c) and APTES-SiO_2_ (d), FTIR diagram of the adsorbents (e) and degumming performance of SiO_2_ and APTES-SiO_2_ adsorbents (f).

The N_2_ adsorption-desorption isotherms of SiO_2_ and APTES-SiO_2_ are shown in Fig. 2a and b. According to the IUPAC classification, both SiO_2_ and APTES-SiO_2_ exhibit typical type IV nitrogen adsorption-desorption curves with an obvious H3 type hysteresis loop, indicating that both samples possess a mesoporous pore structure (G. Zhang et al., 2019). Fig. 2b presents the pore size distribution of the two samples, which predominantly ranges between 7 and 20 nm. In addition, both samples have similar specific surface areas and average pore sizes, centering at approximately 165 m^2^/g and 18 nm, respectively (Table S2). These results suggest that the differences in degumming performance between SiO_2_ and APTES-SiO_2_ are primarily attributable to their distinct chemical structures (Liu et al., 2020). Particle size distribution analysis (Fig. S3) reveals that both SiO_2_ and APTES-SiO_2_ display a normal distribution. However, the latter exhibits a narrower size range of 15–30 μm (compared with 10–40 μm for the pristine SiO_2_) and a larger mean particle size, indicating that APTES modification not only increases the particle size but also improves the size uniformity.

FTIR spectra of SiO_2_ and APTES-SiO_2_ are shown in Fig. 2c. Both samples exhibited identical infrared absorption peaks at 1108, 805, and 472 cm^−1^, which correspond to the bending vibration, symmetric stretching vibration, and asymmetric stretching vibration of Si-O-Si linkages, respectively (Feng et al., 2024). The broad absorption band of SiO_2_ appearing around 3452 cm^−1^ is attributed to physically adsorbed water molecules or the stretching vibration of surface hydroxyl groups (O—H), while the characteristic peak at 969 cm^−1^ corresponds to the bending vibration of Si-OH bonds (Pan et al., 2025). After APTES modification, a reduction in the intensity of the Si-OH absorption band was observed, indicating that APTES consumed a portion of the Si-OH groups on the SiO_2_ surface. Additionally, the band at 698 cm-1 can be assigned to the bending vibration of -CH groups (Luján Ferreira, Pedernera and Esperanza Adrover, 2024) and the characteristic bands of 1562 and 1490 cm^−1^ can be ascribed to stretching vibrations of N—H (Suwan et al., 2023), further confirming APTES molecule grafted onto the SiO_2_ surface. .

The APTES molecule contains ethoxy groups (Si-O-CH_2_CH_3_) and an amino group (−NH_2_). The Si-O-CH_2_CH_3_ groups of APTES can react with the Si-OH groups on the silica surface. After the hydrophilic hydroxyl groups are replaced, new Si-O-Si-(CH_2_)_3_-NH_2_ groups are formed on the surface. Typically, surfaces with a water contact angle (CA) below 90^o^ are considered hydrophilic, whereas those with a CA above 90^o^ are considered hydrophobic (Jayaramulu et al., 2019; W. Wang et al., 2022). As shown in Fig. 2d, APTES-SiO_2_ exhibits a contact angle of 96.12^o^, indicating a hydrophobic surface, while SiO_2_ shows a contact angle of 67.96^o^ (Fig. 2e), indicating a hydrophilic surface. These results suggest that APTES modification alters the surface wettability of SiO_2_ from hydrophilic to hydrophobic, which can promote the dispersion of the SiO_2_ based degumming adsorbent in the oil phase.

### 3.3 Dephosphorization rate of SiO_2_ and APTES-SiO_2_

The adsorption capacity comparison of SiO_2_ and APTES-SiO_2_ for degumming crude rapeseed oil is presented in Fig. 2f. The phospholipid removal rate of SiO_2_ was 69.8%, whereas that of APTES-SiO_2_ reached as high as 98.7%. These data indicate that amino functionalization of silica effectively enhances its adsorption capacity for phospholipids. In addition, the total phenol retention rates of SiO_2_ and APTES-SiO_2_ were 89.1% and 92.3%, respectively. Notably, the total phenol retention rate of the oil degummed with APTES-SiO_2_ is higher than that with SiO_2_, suggesting that APTES-SiO_2_ exhibits stronger selectivity toward phospholipid adsorption. Although phospholipids and total phenols differ in molecular size, our N_2_ adsorption-desorption results showed that the pore structure of APTES-SiO_2_ remained nearly unchanged compared with SiO_2_. Nevertheless, the APTES-SiO_2_ exhibited different selectivity toward total phenols after adsorption, indicating that the adsorption selectivity was not governed by the pore structure or molecular size effects. Instead, the difference in adsorption selectivity was mainly attributed to the change in adsorbent polarity. The modified adsorbent became less polar as shown in Fig. 2, which reduced its affinity for the relatively polar phenolic compounds, thereby resulting in a higher retention rate of total phenols. Overall, APTES-SiO_2_ improves both the phospholipid removal rate and the total phenol retention rate during crude rapeseed oil degumming. This high degumming efficiency can be attributed to the altered surface chemical properties of silica resulting from amino functionalization.

To highlight the advanced degumming performance of the APTES-SiO_2_ adsorbent, a comparison with state-of-the-art SiO_2_ based adsorbents reported in the literature is provided in Table 1. For crude rapeseed oil with a high initial phospholipid content (> 3000 mg/kg), APTES-SiO_2_ exhibited the lowest residual phospholipid content after degumming (45 mg/kg). Moreover, the dephosphorization rate achieved in our work (98.7%) is the highest among all reported SiO_2_ based adsorbents for rapeseed oil degumming. However, due to variations in adsorption conditions across different studies, direct comparisons of adsorption performance are difficult. Therefore, the unit dephosphorization capacity calculated per gram of adsorbent serves as the most appropriate parameter for evaluating adsorbent performance. The unit dephosphorization capacity of the APTES-SiO_2_ adsorbent (346.9 mg/g_adsorbent_) is the highest compared to the other reported SiO_2_ adsorbents as displayed in Table 1.

**Table 1:** Comparison of adsorption performance of SiO_2_ and APTES-SiO_2._

Adsorption material	Adsorption condition	Content of phospholipid in rapeseed oil (mg/kg)	Phospholipid content after adsorption (mg/kg)	Dephosphorization rate (%)	Unit dephosphorusation capacity (mg/g_adsorbent_)	Reference
SiO_2_	45 °C, 30 min	3260	1032.8 ± 101.4	69.8 ± 1.0	238.2 ± 11.8	This work
APTES-SiO_2_	45 °C, 30 min	3260	45.0 ± 13.2	98.7 ± 0.5	346.9 ± 44.7	This work
Silica powder	35 °C, 20 min	2600	390	85.0	221.0	(Yao et al., 2020)
Silicon dioxide	40 °C, 30 min	4020	723	82.0	96.9	(W. Wang et al., 2022)
Iminodisuccinic acid (IDS)-silica	60 °C, 10 h	488.2 (mg/L)	82.5 (mg/L)	83.0	40.5	(Lv et al., 2024)
P6 bleaching earth	28 ± 3 °C, 30 min	110.0 ± 16	10.1	90.9	0.50	(Łaska-Zieja et al., 2020)
Magnesium silicate (MS)	40 °C, 30 min	4180	830	80.2	11.2	(Wang et al., 2023)

### 3.4 APTES-SiO_2_ dosage on the effects of dephosphorization rate, phospholipid content, total phenol retention rate, and total phenol content

**Fig. 3:**
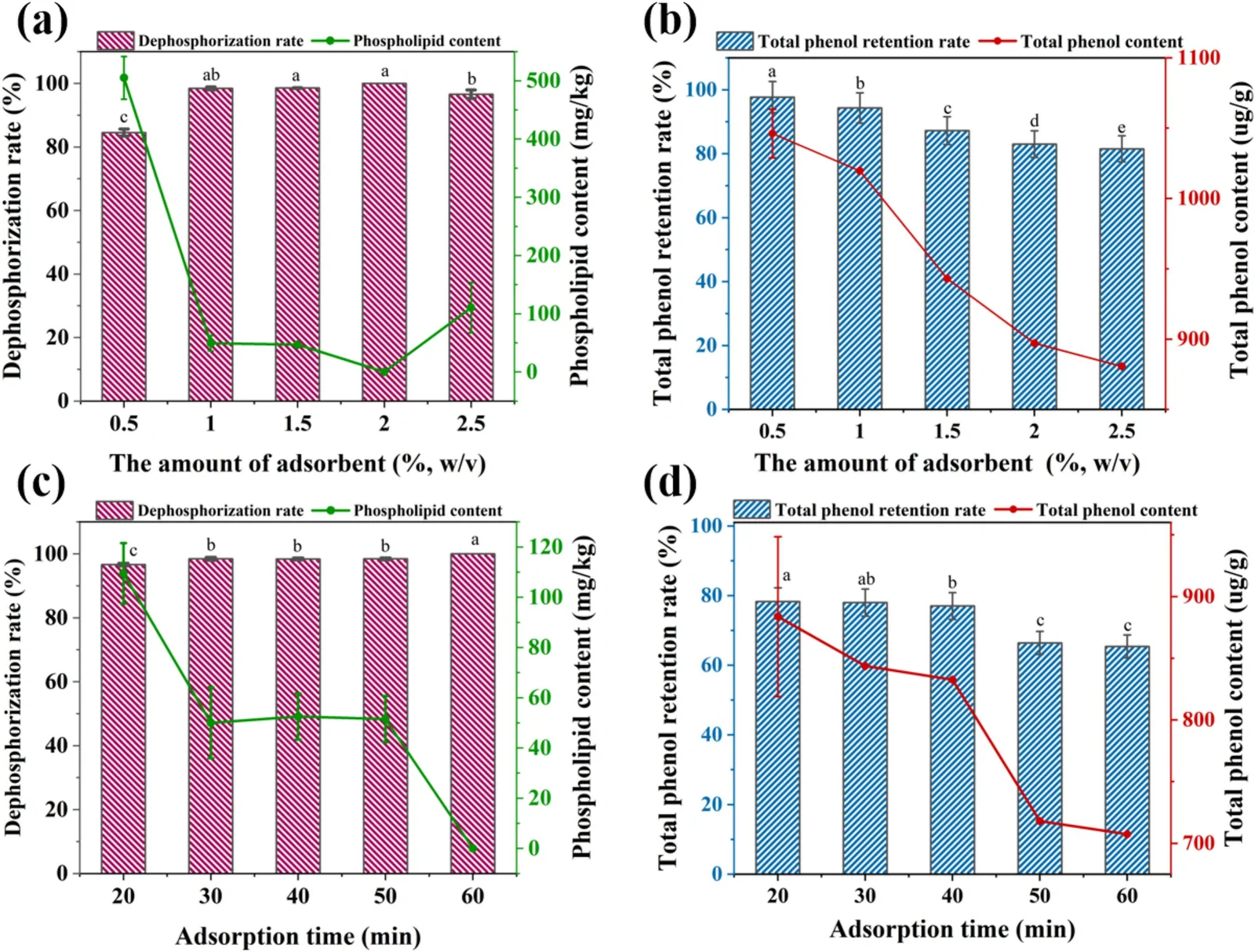
(a) Effects of APTES-SiO_2_ dosage on the phospholipid content. (b) Effects of APTES-SiO_2_ dosage on the total phenolic content. (c) Effects of adsorption time on the phospholipid content. (d) Effects of adsorption time on the total phenolic content.

To optimize the adsorption performance, the researchers investigated the adsorption parameters, namely the dosage of APTES–SiO_2_ (0.5–2.5 g) and the degumming time (20–60 min). As shown in Fig. 3a, when the APTES–SiO_2_dosage increased from 0.5 g to 1 g, the phospholipid removal efficiency exhibited a significant upward trend, which could be attributed to the increase in the total amount of adsorbent, leading to a greater number of adsorption sites. However, when the dosage further exceeded this optimal level, the phospholipid removal efficiency showed a declining trend. This indicates that the adsorption process of phospholipids had approached saturation or that competitive adsorption occurred within the system, thereby reducing the adsorption efficiency for phospholipids.Similar results were obtained in reference (Mu et al., 2025). Furthermore, a higher particle concentration intensifies particle collisions and flocculation, causing agglomeration of the adsorbent and a consequent decrease in the effective specific surface area. The close packing of adsorbent particles prolongs the internal diffusion path for phospholipid molecules, making it difficult for them to diffuse into the internal pore channels and thus diminishing the adsorption efficiency. In addition, as the APTES–SiO_2_ dosage increased, the retention rate of total phenolics in rapeseed oil exhibited a continuous downward trend. This suggests that competitive adsorption between phospholipids and phenolic compounds occurs on the surface of the modified silica. When the dosage reached 1 g, the phospholipid content almost ceased to decrease further, while the retention of total phenolics remained at a relatively high level, indicating that the optimal dosage of APTES–SiO_2_ is 1 g.

The effect of adsorption time on the removal of phospholipids from rapeseed oil is displayed in Fig. 3c. The dephosphorization rate of rapeseed oil showed an increasing trend and then tended to be flat with an increase of adsorption time. Differently, in Fig. 3d the retention of total phenols in rapeseed oil showed a gradual decreasing trend. When the adsorption time exceeded 40 min, the content of phenolic compounds appeared to be significantly reduced. Furthermore, when the adsorption time reached 30 min, the phospholipid content decreased significantly while the total phenol content showed no significant reduction, indicating that 30 min represents the optimal adsorption time for APTES-SiO_2_. This phenomenon can be attributed to the following: during the initial stage of the adsorption reaction, the surface adsorption sites of APTES-SiO_2_ are sufficient and unsaturated, which can react rapidly with phospholipids in rapeseed oil. With the prolongation of adsorption time, the phospholipids and other substances in rapeseed oil gradually occupied the adsorption sites, leading to the gradual saturation of the adsorption sites, and the reaction reached dynamic equilibrium (Sampaio et al., 2019).

### 3.5 Evaluation of physicochemical indexes, flavor and safety

Table 2 showed the comparison of basic physicochemical indexes of degummed rapeseed oil. The peroxide value and acid value were expressed the degree of oxidative rancidity of oils, while phenolics reflected certain antioxidant properties (Yang et al., 2024). The phospholipid content, peroxide value, and acid value of the rapeseed oil samples were reduced following the degumming process. These results indicated that the oxides, peroxides, and other impurities in the oil can be effectively adsorbed by SiO_2_ and APTES-SiO_2_. Meanwhile, the peroxide value (reduced to 0.02) and acid value (reduced to 0.89) of the APTES-SiO_2_ degummed oil were respectively lower than that of SiO_2_ degummed oil. The significant reduction of the acid value confirmed that APTES-SiO_2_ can effectively adsorb free fatty acids in crude oil. In summary, APTES-SiO_2_ demonstrated superior degumming performance for impurities in rapeseed oil.

**Table 2:** Peroxide value, acid value, total phenol content and color of rapeseed oil before and after degumming.

Index	Rapeseed oil	SiO_2_ degummed oil	APTES-SiO_2_ degummed oil
Peroxide value/ (g/100 g)	0.21 ± 0.0	0.03 ± 0.0	0.02 ± 0.0
Acid value (mg KOH/g)	1.79 ± 0.0	1.33 ± 0.1	0.89 ± 0.0
Total phenol content/ (ug/g)	1081.00 ± 0.3	963.12 ± 0.1	1019.24 ± 0.1
L*	3.80 ± 0.5	11.57 ± 1.3	13.70 ± 0.2
a*	1.40 ± 0.7	0.36 ± 0.8	0.32 ± 1.3
b*	0.65 ± 0.2	0.48 ± 0.8	1.55 ± 1.9

*Note: The measurement was performed using a white standard plate (L* = 96.49, a* = −0.26, b* = +1.92) as the reference, L*, a*, b* values are color difference meter test values.

Table 2 also presents the color change parameters (L*, a*, b*) of crude rapeseed oil before and after degumming by SiO_2_ and APTES-SiO_2._ The L* values of degummed rapeseed oil were higher than those of crude rapeseed oil, while the a* and b* values were lower compared to those of crude rapeseed oil. Compared with the SiO_2_ degummed oil, the L* value of APTES-SiO_2_ degummed oil was higher (13.58), indicating that the modified silica resulted in a clearer and more transparent oil sample. In addition, the b* value of the APTES-SiO_2_ treated oil was higher, presenting a yellow color (+b*), and the a* value was lower, presenting a green color (−a*). It is generally accepted that improvements in oil color are indicated by an increase in L* value and a decrease in a* value (L. Zhang et al., 2022). Therefore, APTES-SiO_2_ adsorbent achieves a much superior improvement in oil color compared to unmodified SiO_2_.

**Fig. 4:**
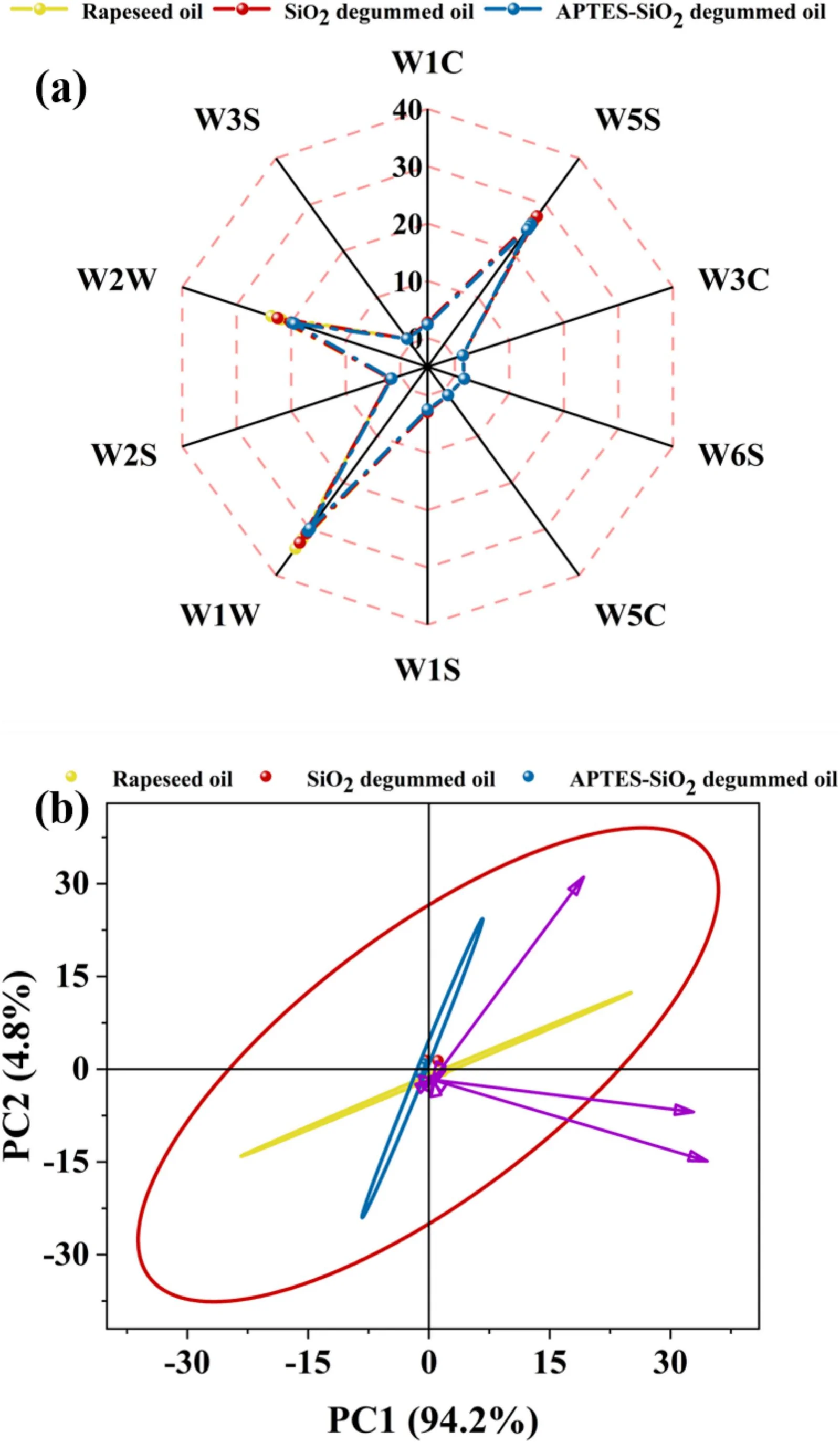
Effect of SiO_2_ and APTES-SiO_2_ on flavor profile of rapeseed oil. (a) Radar chart of electronic nose sensor data. (b) Two-dimensional principal component analysis (PCA) plot of electronic nose sensor data.

The flavor of oils degummed with SiO_2_ and APTES-SiO_2_ was characterized using an electronic nose. In the radargram (Fig. 4a), sensors for hydrogen sulfide (W1W), nitrogen oxides (W5S), and organic sulfur compounds (W2W) showed distinct responses among the three rapeseed oil samples. The W1W sensor exhibited the highest response intensity, with highly similar aroma patterns across samples. Compared with crude oil, both degummed oils showed only slight signal variations, indicating similar flavor profiles, consistent with previous reports (M. Wang et al., 2020). Principal component analysis (PCA) results are presented in Fig. 4b. PC1 and PC2 explained 94.2% and 4.8% of the variance, respectively, with a cumulative variance of 99%, confirming the high reliability of the data (W. Zhang et al., 2023). Compared with crude oil (right cluster), the two degummed oil clusters (left) exhibited subtle spatial variations and were much closer to each other, suggesting no significant differences in overall flavor among the three samples. Thus, APTES-SiO_2_ degumming did not markedly alter the flavor profile relative to crude or SiO₂-degummed oil. Thus, APTES-SiO_2_ degumming did not markedly alter the flavor profile relative to crude or SiO_2_ degummed oil.

**Fig. 5:**
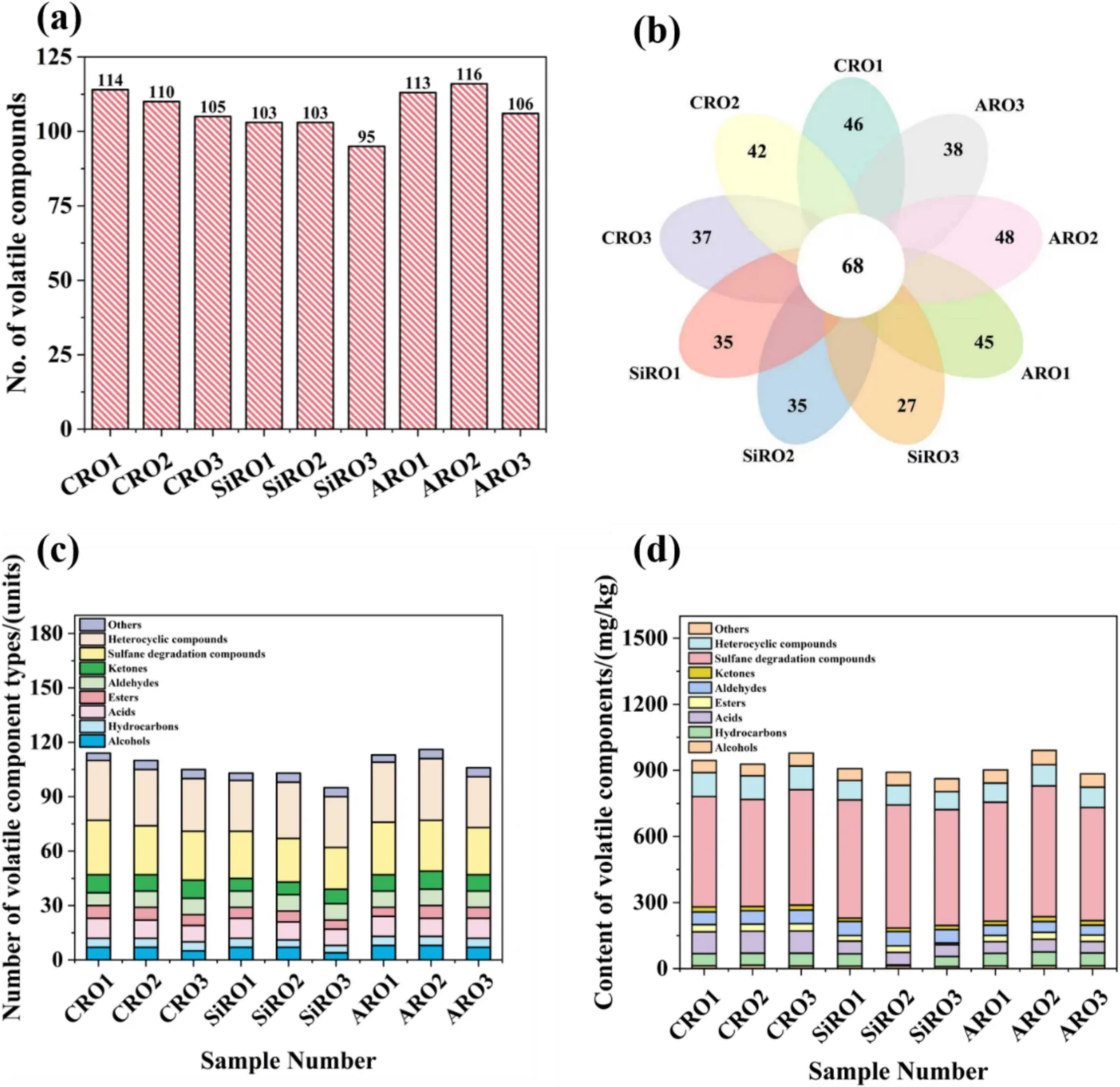
Volatile flavor compounds in rapeseed oil before and after degumming by GC × GC–MS: (a) total numbers, (b) common and differential numbers, (c) number of types, and (d) Content of each type.

To further investigate the flavor characteristics, this study employed comprehensive two-dimensional gas chromatography–mass spectrometry (GC × GC–MS) to analyze 375 volatile components in three groups of rapeseed oil samples: crude rapeseed oil (CRO), silica-degummed oil (SiRO), and APTES-SiO_2_-degummed oil (ARO), with three replicates per group, totaling nine samples. Table S3 presents the qualitative and quantitative results, from which a total of 123 volatile compounds were identified across the three oil types. As shown in Fig. 5a, the number of volatile compounds in CRO ranged from 105 to 114; in SiRO, it decreased to 95–103; while in ARO, it rebounded to 106–116. Thus, the total number of volatile compounds retained in ARO was higher than that in SiRO, indicating that ARO exhibited better preservation of volatile components. Fig. 5b shows that 68 volatile compounds were common to all three groups. Notably, ARO retained more unique volatile components (38–48), a number comparable to that of CRO (37–46). Figs. 5c-d classify the volatile compounds into nine categories, among which glucosinolate degradation products (30 compounds) and heterocyclic compounds (36 compounds) were the main categories. As shown in Figs. 5c-d and supported by Table S3, glucosinolate degradation products and heterocyclic compounds exhibited the highest variety and abundance among all volatile components. Glucosinolate degradation products are primarily generated from the thermal or enzymatic breakdown of glucosinolates during the roasting of rapeseed, yielding characteristic volatile compounds dominated by nitriles, isothiocyanates, and thiocyanates.These compounds are critical contributors to the pungent, sulfurous aroma of rapeseed oil. In all three oil samples, the total content of glucosinolate degradation products was relatively high, ranging from 486.6 to 593.9 mg/kg, with 3-methylcrotononitrile and 5-hexenenitrile being the most abundant and thus the predominant contributors. Additionally, compounds such as 3-butenyl isothiocyanate, cyclopentyl isothiocyanate, and phenylpropionitrile have also been recognized as important odorants imparting pungent and pickled-like notes. Compared with CRO, the total content of these degradation products increased after degumming (in both SiRO and ARO), which is consistent with the findings of Liu et al. (W. Wang et al., 2023).

Heterocyclic compounds are mainly formed via the Maillard reaction and impart nutty, roasted, and caramel-like notes to rapeseed oil. Among these, pyrazines (e.g., 2,3,5-trimethylpyrazine, 2-ethyl-5-methylpyrazine) and furanones (e.g., 2(5H)-furanone) are considered key aroma-active compounds owing to their low odor thresholds and high odor intensities (Y. Zhang et al., 2020). After degumming, the total content of heterocyclic compounds decreased in both treated oils. However, ARO exhibited a smaller loss (86.7–96.3 mg/kg) compared with SiRO (81.5–89.2 mg/kg), indicating that APTES-SiO_2_ degumming better preserves these important flavor components, which is beneficial for maintaining the overall characteristic flavor of rapeseed oil.

Apart from the two dominant flavor categories mentioned above, aldehydes—primarily furfural and 5-hydroxymethylfurfural—contribute bitter almond-like and caramel-like notes. Their contents in ARO were lower than those in CRO, which may be attributed to the adsorption of these polar aldehydes by APTES-SiO_2_; nevertheless, the overall aroma balance remained largely intact. Alcohols, esters, and acids generally possess high odor thresholds or exhibit unpleasant odors, and thus contribute only marginally to the overall flavor profile. Notably, the content of acids, mainly acetic acid, decreased substantially after degumming, which is beneficial for suppressing rancid off-notes. Overall, there were no significant differences in the types and contents of volatile flavor compounds between CRO and ARO, suggesting that the APTES-SiO_2_ degumming process can preserve the original characteristic flavor of rapeseed oil.

To assess whether APTES molecules leach from the adsorbent into rapeseed oil during degumming, potentially compromising oil safety, Fourier transform infrared spectroscopy (FTIR) was performed on the degummed oil. As shown in Fig. S5., crude rapeseed oil exhibited characteristic peaks at 3008 cm^−1^ and 723 cm^−1^, corresponding to the stretching vibrations of the olefinic C=C bond and the cis-substituted C=C bond, respectively (Siudem et al., 2019). The bands at 1380 cm^−1^ and 2857 cm^−1^ were assigned to the bending vibration of CH_2_ groups (Liu, Chen, Shi, Yang and Han, 2020) and the symmetric stretching vibration of aliphatic C—H bonds (Fediuc & Oroian, 2025), while the peaks at 1750 cm^−1^ and 1463 cm^−1^ originated from the stretching vibration of C=O groups in triglycerides (H. Liu et al., 2020). Notably, the FTIR spectra of oils degummed with SiO_2_ and APTES-SiO_2_ were nearly identical to that of crude oil, with no APTES-related groups detected in the degummed oil. These results confirm that APTES does not leach into the degummed oil, thereby ensuring oil safety. This finding further demonstrates that APTES is firmly anchored onto the SiO_2_ surface, leaving no detectable residue in the degummed oil.

### 3.6 Exploring adsorption mechanism of APTES modified silica

#### 3.6.1 Adsorption kinetics

**Fig. 6:**
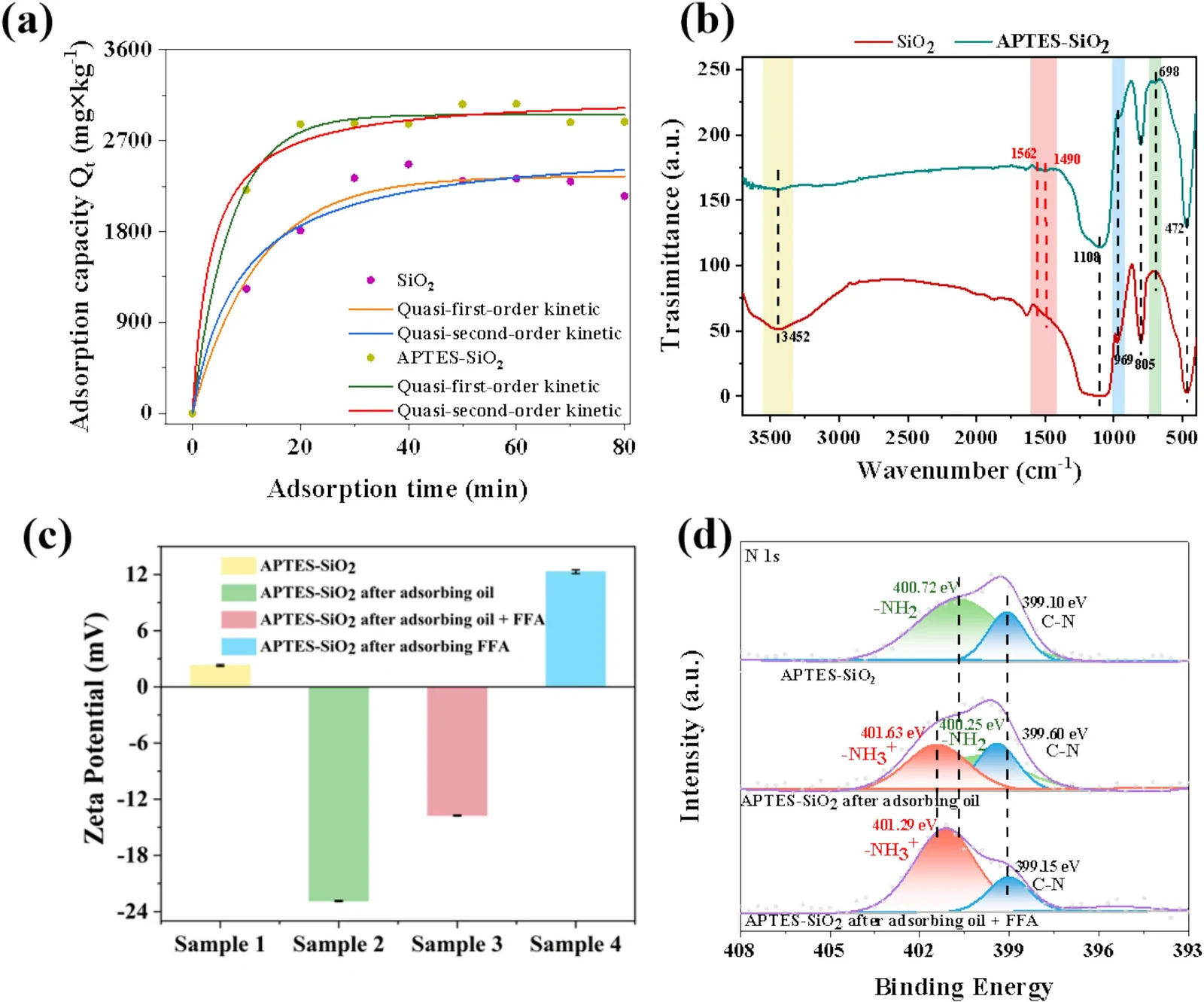
(a) Kinetic curves for the adsorption of phospholipids on SiO_2_ and APTES-SiO_2_. (b) FTIR spectra of APTES-SiO_2_ before and after degumming. (c) Zeta potential of APTES-SiO_2_ before and after adsorption in different media. (d) N1s XPS spectra of APTES-SiO_2_ before and after degumming.

The adsorption kinetics of the two adsorbents were investigated using 300 mL of crude rapeseed oil at 45 °C, as shown in Fig. 6a, with fitting results summarized in Table 3. For pristine SiO_2_, the correlation coefficient of the pseudo-first-order kinetic model (R^2^ = 0.975) was higher than that of the pseudo-second-order model (R^2^ = 0.950), indicating that the adsorption process is primarily governed by physical adsorption. For APTES-SiO_2_, the pseudo-first-order model also exhibited a higher correlation coefficient (R^2^ = 0.994) than the pseudo-second-order model (R^2^ = 0.986), with the former value being closer to unity. Thus, the adsorption kinetics of APTES-SiO_2_ are also better described by the pseudo-first-order model, suggesting a predominantly physical adsorption mechanism. Notably, APTES-SiO_2_ exhibited higher k_1_ and k_2_ adsorption rate constants than pristine SiO_2_, indicating a greater phospholipid adsorption capacity. This difference can be attributed to the additional adsorption sites provided by the interaction between APTES molecules and phospholipids.

**Table 3:** Adsorption kinetic parameters for phospholipids adsorption on SiO_2_ and APTES-SiO_2_.

Adsorption material	Quasi-first-order kinetic model			Quasi-second-order kinetic model		
	q_e_/ (mg/kg)	k_1_/ min	R^2^	q_e_/ (mg/kg)	k_2_/ min	R^2^
SiO_2_	2345.2	0.083	0.975	2676.5	4.233 × 10^−5^	0.950
APTES-SiO_2_	2956.2	0.142	0.994	3154.7	9.069 × 10^−5^	0.986

#### 3.6.2 Exploring the extra adsorption sites of APTES-SiO_2_ by FTIR, zeta potential, and XPS

The structural change of APTES-SiO_2_ after degumming was characterized by FTIR (Fig. 6b). Prior to degumming, APTES-SiO_2_ exhibited symmetric and asymmetric characteristic peaks of -NH_2_ at 3292 cm^−1^ (Ahmed et al., 2013). After degumming, the adsorbent (washed with n-hexane to remove residual oil) showed new characteristic peaks of -NH_3_^+^ at 2932 cm^−1^ and 1521 cm^−1^ (Lujan Ferreira et al., 2024; Q. Zhang et al., 2021), indicating protonation of the amino groups during adsorption. These ammonium ions (-NH_3_^+^) serve as additional adsorption sites for enhanced phospholipid removal.

To further verify this protonation, zeta potential measurements were conducted in neutral phosphate buffer (Fig. 6c). The ζ potential of APTES-SiO_2_ before adsorption was near zero. After adsorption in crude rapeseed oil, it became highly negative (−22.9 mV), due to coverage of the adsorbent surface by phospholipids bearing negatively charged phosphate head groups. Although free fatty acids (FFA, 0.3–0.5%) in crude oil can protonate amino groups, this effect is masked by the abundant adsorbed negative phospholipids. When 10 mL of oleic acid (as a simulated FFA) was added to 30 mL of crude oil, the ζ potential of the recovered adsorbent increased (though remained negative at −13.7 mV) (Ishizaki et al., 2021). To exclude interference from other oil components, APTES-SiO_2_ was dispersed in pure oleic acid; the ζ potential turned positive (12.3 mV), definitively confirming protonation of -NH_2_ to -NH_3_^+^ (Darpentigny et al., 2020). Thus, the amino groups grafted on silica are protonated by free fatty acids in crude oil, forming positively charged ammonium groups.

XPS analysis of the N 1 s region further supported this conclusion (Fig. 6d; full spectra in Fig. S6). Before adsorption, the N 1 s spectrum of APTES-SiO_2_ showed two fitted peaks at ∼399.1 eV (C—N) and ∼ 400.7 eV (-NH_2_) (Mohamed Mohsin et al., 2019). After phospholipid adsorption, a new peak appeared at 401.6 eV, assigned to -NH_3_^+^, while the -NH_2_ peak weakened (Y. Liu et al., 2022). Upon addition of oleic acid to crude oil, the -NH_2_ peak disappeared and the -NH_3_^+^ peak at 401.29 eV significantly increased, confirming that free fatty acids induce amino protonation, consistent with the zeta potential results.

#### 3.6.3 Proposed adsorption mechanism of new APTES-SiO_2_ adsorbent

In this work, APTES-mediated surface modification endows SiO_2_ with unique physicochemical properties, which can achieve the high efficient adsorption of phospholipids for degumming of crude rapeseed oil. During modification, the polar hydroxyl groups on the silica surface are replaced by alkyl chains bearing amino groups. The hydrocarbon chain (e.g., propyl group) acts as a hydrophobic moiety, improving the dispersion of APTES-SiO_2_ in rapeseed oil and thereby increasing the accessibility of the adsorbent to phospholipid molecules. Furthermore, the two hydrophobic fatty acid tails of phospholipids undergo hydrophobic association with the hydrophobic groups on the modified silica surface, accompanied by dehydration. This process increases the entropy of the adsorption system, reduces the Gibbs free energy, and promotes spontaneous adsorption under mild conditions (Puddu & Perry, 2012).

**Fig. 7:**
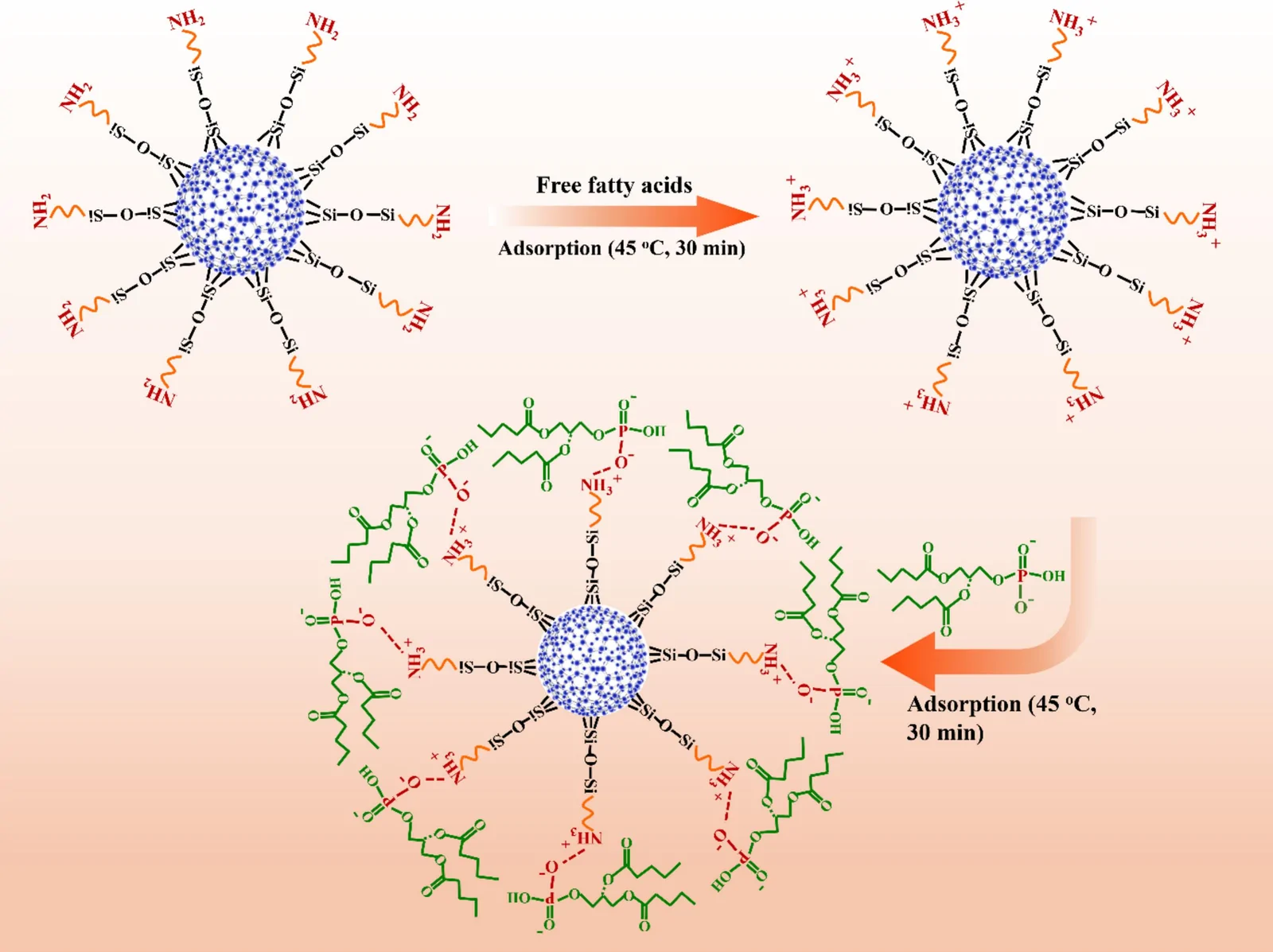
Schematic illustration of possible adsorption mechanism between phosphatides and APTES-SiO_2_.

On the other hand, the grafted amino groups with hydrocarbon chain via Si-O-Si onto the surface of silica can be protonated in the presence of free fatty acid as demonstrated by FTIR, zeta potential, and XPS results. As proposed in Fig. 7, the protonated amino groups (-NH_3_^+^) exhibited positive charge distributed onto the surface of APTES-SiO_2_. It was known for us that the phospholipid head contained negatively charged phosphate groups, such as-OPO_3_^2−^ and -PO_4_^2−^, which can attract the positively charged amino group via a strong Coulombic electrostatic interaction, thereby achieving efficient and selective adsorption of phospholipids onto APTES-SiO_2_ adsorbent. Therefore, it was reasonable to conclude that the protonated amino groups provided new and extra adsorption sites compared to that of unmodified SiO_2_, which thus promoted the removal rate of phospholipids for degumming rapeseed crude oil.

### 3.7 Degumming stability and degumming expansion

**Fig. 8:**
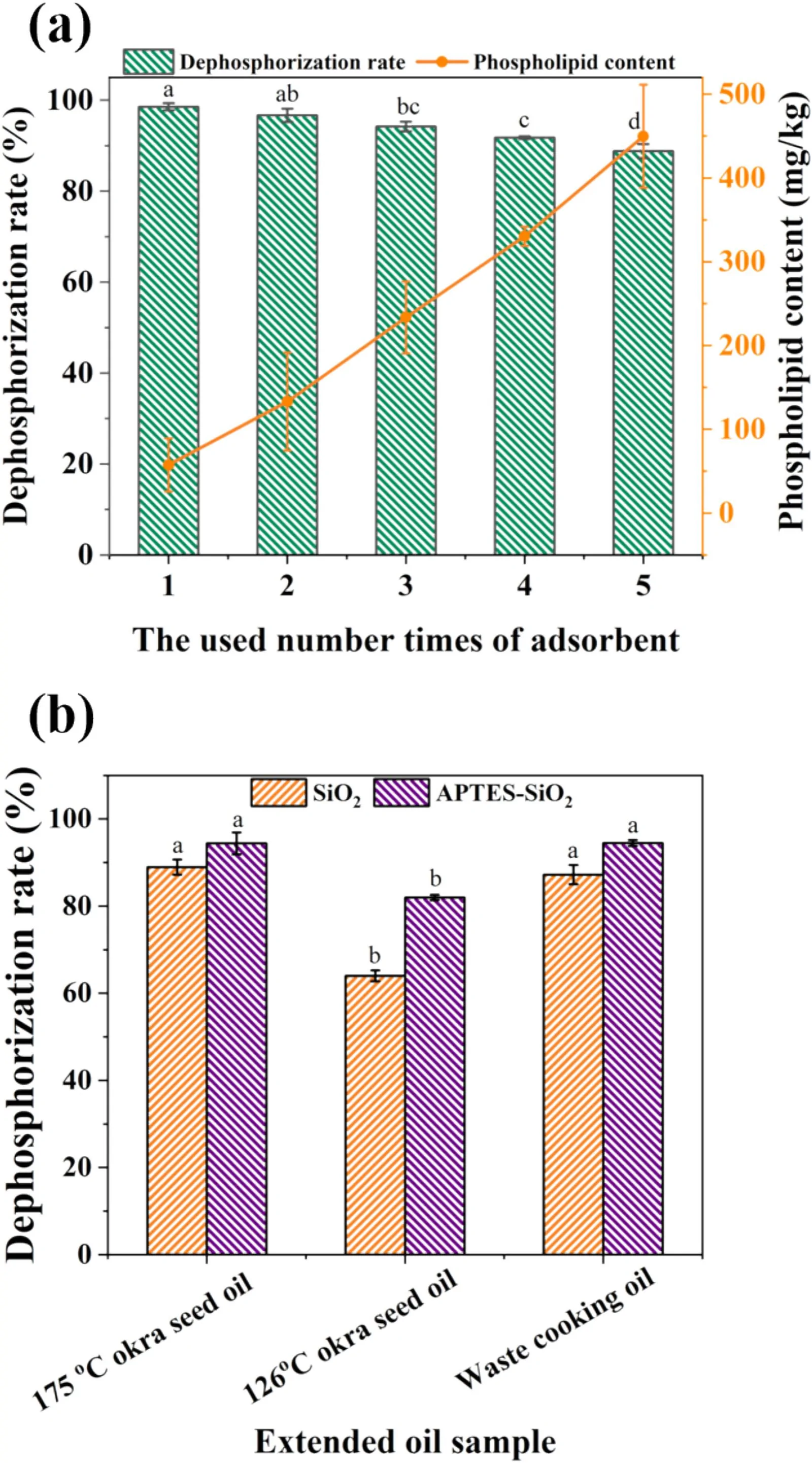
(a) The reusability of APTES-SiO_2_ in rapeseed oil. (b) Effects of SiO_2_ and APTES-SiO_2_ on the phospholipid content from crude okra seed oil and waste cooking oil extracted at different temperatures.

Fig. 8a showed the degumming stability of the APTES-SiO_2_ and Fig. S7 showed the SEM image after adsorbing phospholipid. It can be found that there is no the change of morphological features. After each adsorption cycle, the adsorbent was separated by centrifugation and rinsed repeatedly with ultrapure water at 65 °C until no oil remained, following dried in an oven at 90 °C for 15 h. The phospholipid removal rate of APTES-SiO_2_ remained above 90% during the four cycles of rapeseed oil degumming. Although the phospholipid removal rate gradually decreased in subsequent one cycles, the degumming effect was still satisfactory after the fifth cycles, with the phospholipid removal rate remaining above 85%. To expand the potential application of APTES-SiO_2_ adsorbent for removal of phospholipids in the oil refining, Fig. 8b compared the degumming effects on crude okra seed oil and the waste cooking oil. It can be observed that the phospholipid removal efficiency of APTES-SiO_2_ was significantly higher than that of unmodified SiO_2_. For example, okra seed crude oil at 126 °C and 175 °C, the phospholipid removal rate reached 82.0% and 94.4%, respectively. Similarly, for the degumming of waste cooking oil, APTES-SiO_2_ showed much higher degumming performance than that of SiO_2_ (94.5% vs 87.2%, respectively). The above results indicated that APTES-SiO_2_ adsorbent not only can remove the phospholipids from rapeseed and crude okra seed oil but also from waste cooking oil, exhibiting a certain degree of universality for degumming in the oil refining.

## 4 Conclusion

In summary, an amino-modified silica (APTES-SiO_2_) was prepared and applied for the adsorption of phospholipids from crude rapeseed oil under mild conditions. The results showed that the phospholipid removal rate of APTES-SiO_2_ was as high as 98.8%, and the total phenol retention rate can reach 92.3%. Meanwhile, the peroxide value and acid value of the oil product decreased and the color improved. Moreover, there was little change of the flavor after degumming for APTES-SiO_2_ adsorbent. The optimized degumming conditions were at 45 °C for 30 min with 1 g addition of adsorbent. The prepared APTES-SiO_2_ adsorbent exhibited the hydrophobic surface, which enhanced the dispersion of adsorbent in rapeseed oil and thus increased the accessibility of the adsorbent to phospholipid molecules. In addition, the protonated amino groups provided new and extra adsorption sites, resulting in Coulombic electrostatic interaction with negatively charged phosphate molecules, which thus promoted the removal rate of phospholipids. Our findings provided an example of rational design and preparation of highly dispersed and efficient adsorbent for degumming crude oil.

## CRediT authorship contribution statement

**Meihong Wu:** Writing – review & editing, Investigation, Data curation. **Ting Mo:** Writing – original draft, Methodology, Data curation. **Maoting Chen:** Investigation, Data curation. **Shuang Wang:** Methodology, Data curation. **Qiao Dai:** Investigation. **Tongwei Guan:** Resources. **Changsheng Liu:** Writing – review & editing. **Loubna Arab:** Writing – review & editing. **Xuemei Liao:** Writing – review & editing, Supervision, Resources, Project administration, Funding acquisition, Conceptualization. **Wenlin Li:** Writing – review & editing, Resources, Project administration, Funding acquisition.

## Declaration of competing interest

The authors declare that they have no known competing financial interests or personal relationships that could have appeared to influence the work reported in this paper.

## Data Availability

Data will be made available on request.

## Appendix A Supplementary data

Supplementary materia: Supplementary data include the standard curves for the molybdenum blue colorimetric method (AOCS Official Method Ca 12-55) and the Folin–Ciocalteu method; SEM, EDS, particle-size distribution, FTIR, and XPS analyses of SiO₂ and APTES-SiO₂ before and after phospholipid adsorption/degumming ; and comparisons of phospholipid determination methods, textural properties of SiO₂ and APTES-SiO₂, and GC×GC–MS identification of volatile components in rapeseed oil before and after degumming.
