# Supplementary material for: Highly efficient removal of phospholipids in the degumming of crude rapeseed oil using APTES-modified silica adsorbent
Source: Food Chem X. 2026 Sep 3;39:104407. doi: 10.1016/j.fochx.2026.104407 (PMC13578962; doi:10.1016/j.fochx.2026.104407)
Supplement: Supplementary file 1 [file mmc1.docx]

**Highly efficient removal of phospholipids in the degumming of crude rapeseed oil using APTES-modified silica adsorbent**

Meihong Wu ^a^, Ting Mo ^a^, Maoting Chen ^a^, Shuang Wang ^a^, Qiao Dai ^a^, Tongwei Guan ^a^, Changsheng Liu ^b^, Loubna Arab ^d^, Xuemei Liao ^a, c, *^, Wenlin Li ^b, *^

*^a^ School of Food and Biological Engineering, Xihua University, 610039, Chengdu, China*

*^b^ Oil Crops and Lipids Process Technology National & Local Joint Engineering Laboratory, Key Laboratory of Oilseeds Processing, Ministry of Agriculture and Rural Affairs, Oil Crops Research Institute, Chinese Academy of Agricultural Sciences, Wuhan430062, China*

*^c^ Yibin Xihua University Research Institute, Yibin, 644001, China*

*^d^ Laboratory of Membrane Processes and Techniques of Separation and Recovery (LPMTSR), Faculty of Technology, Abderrahmane Mira University of Bejaia, 06000, Algeria.*

*Corresponding Author E-mail: [xmliao@mail.xhu.edu.cn](mailto:xmliao@mail.xhu.edu.cn) (X. Liao) ; wenlinli2005@163.com (W. Li)

**Fig. S1.** (a) Standard curve of molybdenum blue colorimetric method (AOCS Official Method Ca 12-55, 2009), (b) Standard curve of Folin-Ciocalteu colorimetric Method.

**Fig. S2.** SEM images of SiO_2_ (a and b), APTES-SiO_2_ (c and d).

**Fig. S3. Particle size distribution diagrams of SiO_2_ (a) and APTES-SiO_2_ (b).**

**Fig. S4. EDS Energy dispersive spectroscopy of SiO_2_.**

**Fig. S6.** FTIR spectra of degummed rapeseed oil by SiO_2_ and APTES-SiO_2_.

**Fig. S7.** XPS survey spectrum of APTES-SiO_2_ before and after degumming.

**Fig. S8.** SEM images of APTES-SiO_2_ after adsorbing phospholipids from rapeseed oil.

**Table S1.** Comparison of methods for determining phospholipid content.

| sample | Test method | Phospholipid content (mg/kg) |
| --- | --- | --- |
| Rapeseed oil | Acetone extraction gravimetry | 3260 |
|  | Molybdenum blue colorimetry | 3391 |

**Table S2.** Specific surface area, pore volume and average pore diameter of SiO_2_ and APTES-SiO_2_.

| Sample | Specific surface area/（m^2^･g^-1^） | Pore volume/（cm^3^･g^-1^） | aperture/ (nm) |
| --- | --- | --- | --- |
| SiO_2_ | 167 | 0.768 | 18.4 |
| APTES-SiO_2_ | 164 | 0.733 | 17.8 |

**Table S3.** The identification results of volatile components in rapeseed oil before and after degumming treatment with different adsorbents by GC×GC-MS were obtained.

| Classification | | Retention Time/(min) | | GAS No. | Compounds | Content of main volatile components/(mg/kg) | | | | | | | | |
| --- | --- | --- | --- | --- | --- | --- | --- | --- | --- | --- | --- | --- | --- | --- |
|  |  |  |  |  |  | CRO1 | CRO2 | CRO3 | SiRO1 | SiRO2 | SiRO3 | ARO1 | ARO2 | ARO3 |
| Alcohols | | 19.000 | | 513-85-9 | 2,3-Butanediol | 0.858 | 1.703 | 1.280 | ND | ND | ND | 0.984 | 0.633 | 1.243 |
|  |  | 32.533 | | 36653-82-4 | 1-Hexadecanol | ND | 0.215 | ND | 0.253 | ND | ND | 0.486 | 0.257 | 0.351 |
|  |  | 13.733 | | 19780-84-8 | 5-HEXYN-3-OL | 0.928 | 1.176 | 1.243 | 1.281 | 1.283 | 1.222 | 1.054 | 1.466 | 1.112 |
|  |  | 21.600 | | 98-00-0 | Furfuryl alcohol | 8.915 | 8.734 | 7.853 | 6.964 | 7.016 | 6.427 | 6.703 | 8.375 | 7.695 |
|  |  | 14.127 | | 50551-88-7 | 5-methylhex-5-en-2-ol | ND | ND | ND | ND | 1.167 | 1.011 | 1.094 | ND | 0.938 |
|  |  | 27.000 | | 60-12-8 | Phenethyl alcohol | 1.598 | 1.518 | 1.499 | 1.344 | 1.339 | 1.198 | 1.235 | 1.311 | 1.569 |
|  |  | 10.193 | | 123-51-3 | Isoamyl alcohol | 0.136 | ND | 0.159 | 0.112 | 0.109 | ND | ND | 0.118 | 0.121 |
|  |  | 25.533 | | 83-46-5 | beta-Sitosterol | 0.252 | 1.313 | ND | 1.284 | 1.086 | ND | 0.870 | 0.930 | ND |
|  |  | 22.000 | | 143-08-8 | 1-Nonanol | 0.611 | 0.546 | ND | 0.554 | 0.538 | ND | 0.424 | 0.509 | ND |
|  | | | **Total** | | | 13.298 | 15.205 | 12.034 | 11.792 | 12.538 | 9.858 | 12.850 | 13.597 | 13.028 |
| Hydrocarbons | | 33.867 | | 13330-65-9 | 2,4-Dimethoxyphenol | 0.298 | 0.206 | 0.272 | 0.272 | 0.375 | ND | 0.218 | 0.242 | 0.274 |
|  |  | 34.867 | | 544-76-3 | Hexadecane | 1.664 | 1.720 | 1.902 | 1.699 | 1.665 | 1.614 | 1.527 | 1.579 | 1.569 |
|  |  | 32.533 | | 7786-61-0 | 4-Vinylguaiacol | 1.157 | 1.368 | 1.502 | 1.827 | 1.693 | 1.371 | 1.922 | 1.485 | 1.456 |
|  |  | 30.067 | | 111-02-4 | Squalene | 51.664 | 51.556 | 54.096 | 51.501 | ND | 41.786 | 52.702 | 58.420 | 54.231 |
|  |  | 36.333 | | 14059-92-8 | Phenol, 4-ethyl-2,6-dimethoxy- | 0.276 | 0.303 | 0.306 | 0.454 | 0.430 | 0.518 | 0.301 | 0.392 | 0.455 |
|  | | | **Total** | | | 55.060 | 55.153 | 58.079 | 55.752 | 4.163 | 45.289 | 56.670 | 62.117 | 57.985 |
| Acids | | 23.533 | | 1883-13-2 | 3-hydroxy Lauric Acid | 0.884 | 1.034 | 0.610 | 1.096 | 0.558 | 0.827 | 0.474 | 0.786 | 0.630 |
|  |  | 21.793 | | 4536-23-6 | 2-Methylhexanoic acid | 0.937 | 0.819 | 1.300 | 1.022 | 1.028 | ND | 1.128 | 1.077 | 1.071 |
|  |  | 24.800 | | 591-80-0 | 4-Pentenoic acid | 31.401 | 29.762 | 30.897 | 30.881 | 31.623 | 30.139 | 27.790 | 28.155 | 22.443 |
|  |  | 26.800 | | 1577-22-6 | 5-Hexenoic Acid | 18.241 | 18.414 | 18.189 | 17.031 | 16.874 | 16.190 | 11.990 | 14.394 | 12.582 |
|  |  | 19.400 | | 79-31-2 | Isobutyric acid | 0.891 | 0.988 | ND | 0.796 | 0.814 | ND | 0.618 | 0.690 | 0.615 |
|  |  | 20.800 | | 109-52-4 | Pentanoic acid | 0.277 | 0.255 | 0.235 | 1.000 | 0.964 | 1.035 | 0.757 | 0.819 | 0.788 |
|  |  | 44.467 | | 57-10-3 | Palmitic acid | 0.260 | 0.172 | 0.196 | 0.430 | 0.592 | 0.569 | 0.764 | 0.769 | 0.758 |
|  |  | 41.393 | | 693-71-0 | 13-Octadecenoic acid, (13E)- | 0.090 | ND | ND | 0.621 | ND | 0.518 | 0.365 | ND | 0.397 |
|  |  | 16.333 | | 64-19-7 | Acetic acid | 40.375 | 43.082 | 44.055 | 0.262 | 0.276 | 0.264 | 6.433 | 8.148 | 9.867 |
|  |  | 18.600 | | 79-09-4 | Propionic acid | 3.164 | 3.209 | 3.103 | 2.570 | 2.534 | 2.459 | 1.525 | 1.458 | 1.711 |
|  |  | 25.600 | | 142-62-1 | Hexanoic acid | 1.876 | 1.729 | 1.678 | 1.714 | 1.941 | 1.421 | 1.483 | 1.188 | 1.379 |
|  | | | **Total** | | | 98.394 | 99.464 | 100.263 | 57.423 | 57.205 | 53.422 | 53.328 | 57.484 | 52.240 |
| Esters | | 26.660 | | 39726-36-8 | 1,6-anhydro-2,3-dideoxy-beta-d-erythro-hexopyranose | 0.785 | 0.878 | ND | ND | ND | ND | ND | 0.898 | 0.784 |
|  |  | 21.000 | | 104-61-0 | (±)-γ-Nonalactone | 5.142 | 4.522 | 3.430 | 2.898 | 2.995 | 3.379 | 3.576 | 4.189 | 3.778 |
|  |  | 8.267 | | 818-57-5 | 4-Pentenoic Acid Methyl Ester | 0.065 | 0.048 | 0.067 | 0.046 | 0.045 | 0.045 | 0.148 | 0.153 | ND |
|  |  | 31.460 | | 4430-36-8 | ERUCIN | 0.159 | 0.136 | 0.188 | 0.208 | 0.200 | 0.205 | ND | 0.142 | 0.188 |
|  |  | 29.533 | | 823-22-3 | delta-Hexalactone | 22.242 | 22.061 | 24.144 | 16.531 | 20.726 | ND | 19.796 | 21.850 | 21.004 |
|  |  | 28.800 | | 124-10-7 | Methyl Myristate | 3.414 | 3.467 | 3.748 | 3.163 | 3.010 | 2.857 | 2.382 | 3.185 | 2.873 |
|  |  | 21.467 | | 112-62-9 | Methyl Oleate | 2.094 | 2.255 | 2.175 | 2.189 | 2.827 | 1.550 | 1.784 | 1.822 | 1.493 |
|  | | | **Total** | | | 33.117 | 32.489 | 33.751 | 25.035 | 29.804 | 8.036 | 27.686 | 31.342 | 29.336 |
| Aldehydes | | 18.467 | | 4313-03-5 | (E,E)-2,4-Heptadienal | ND | 2.258 | 2.835 | 2.539 | 1.980 | 1.421 | 1.979 | 2.502 | 1.198 |
|  |  | 23.933 | | 2463-77-6 | 2-Undecenal | 0.356 | 0.284 | 0.350 | 0.406 | 0.342 | 0.368 | 0.311 | 0.285 | 0.237 |
|  |  | 37.667 | | 67-47-0 | 5-Hydroxymethylfurfural | 12.576 | 11.986 | 13.927 | 15.183 | 14.654 | 14.539 | 3.088 | 2.897 | 3.249 |
|  |  | 6.267 | | 123-73-9 | Crotonaldehyde | 0.445 | 0.480 | 0.459 | 0.500 | 0.514 | 0.521 | 0.416 | 0.404 | 0.407 |
|  |  | 18.600 | | 100-52-7 | analytical standard | ND | 0.595 | 0.887 | 0.700 | 0.627 | 0.779 | 0.617 | 0.531 | 0.600 |
|  |  | 21.867 | | 3913-81-3 | 3-Heptylacrolein | 1.175 | 1.156 | 1.211 | 1.147 | 1.091 | 1.245 | 0.960 | 1.011 | 0.910 |
|  |  | 16.867 | | 98-01-1 | Furfural | 39.846 | 40.799 | 38.716 | 42.026 | 42.472 | 40.323 | 36.185 | 39.169 | 36.185 |
|  |  | 16.000 | | 124-19-6 | Nonanal | 2.157 | 2.558 | 3.356 | 1.699 | 2.636 | 1.321 | 2.753 | 1.804 | 1.730 |
|  |  | 7.133 | | 66-25-1 | Hexanal | 0.419 | 0.424 | 0.377 | 0.420 | 0.452 | 0.359 | 0.377 | 0.422 | 0.378 |
|  | | | **Total** | | | 56.973 | 60.541 | 62.116 | 64.620 | 64.768 | 60.877 | 46.685 | 49.024 | 44.893 |
| Ketones | | 41.133 | | 17016-83-0 | (4S)-(-)-4-Isopropyl-2-oxazolidinone | 0.242 | 0.265 | 0.341 | 0.454 | 0.358 | 0.399 | 0.304 | 0.358 | 0.333 |
|  |  | 18.927 | | 1575-57-1 | 2-oxobutyl acetate | 1.301 | 0.646 | 1.042 | ND | 0.875 | 1.038 | ND | 0.968 | 1.002 |
|  |  | 6.600 | | 600-14-6 | 2,3-Pentanedione | 0.467 | 0.449 | 0.502 | 0.403 | 0.341 | 0.440 | 0.436 | 0.436 | 0.378 |
|  |  | 9.660 | | 1120-72-5 | 2-Methylcyclopentanone | 0.185 | 0.308 | 0.430 | 0.203 | ND | 0.230 | ND | ND | 0.106 |
|  |  | 31.927 | | 19444-84-9 | α-Hydroxy-γ-butyrolactone | ND | 0.558 | 0.497 | ND | ND | ND | 0.463 | 0.377 | ND |
|  |  | 25.333 | | 765-70-8 | 3-Methyl-1,2-cyclopentanedione | 2.452 | 2.362 | 2.288 | 1.904 | 1.989 | 2.019 | 1.849 | 2.218 | 2.098 |
|  |  | 19.933 | | 930-60-9 | cyclopent-4-ene-1,3-dione | 0.625 | 0.671 | 0.758 | 0.317 | 0.281 | ND | 0.287 | 0.453 | 0.369 |
|  |  | 9.467 | | 120-92-3 | Cyclopentanone | 0.091 | 0.074 | 0.057 | 0.075 | 0.066 | 0.069 | 0.033 | 0.044 | 0.045 |
|  |  | 12.467 | | 116-09-6 | Hydroxyacetone | 13.220 | 13.737 | 12.621 | 11.572 | 11.940 | 11.756 | 12.270 | 14.214 | 12.856 |
|  |  | 22.267 | | 1725-03-7 | oxacyclododecan-2-one | 0.306 | ND | ND | ND | ND | ND | 0.110 | 0.094 | ND |
|  |  | 17.200 | | 592-20-1 | Acetoxy-2-propanone | 4.335 | ND | 3.579 | ND | ND | 2.425 | 2.427 | 2.897 | 2.779 |
|  | | | **Total** | | | 23.224 | 19.069 | 22.115 | 14.928 | 15.850 | 18.379 | 18.179 | 22.061 | 19.967 |
| Glucosinolate degradation products | | 40.467 | | 756486-85-8 | 2-Oxazolidinethione,3-ethenyl- | 0.286 | 0.385 | 0.396 | 0.511 | 0.500 | 0.481 | 0.390 | 0.481 | 0.398 |
|  |  | 27.467 | | 54096-45-6 | 1-cyano-3,4-epithiobutane | 4.284 | 3.580 | 3.853 | ND | ND | ND | 6.000 | 5.987 | 5.265 |
|  |  | 21.667 | | 4426-83-9 | 1-Heptyl isothiocyanate | 0.173 | 0.177 | 0.187 | 0.186 | 0.297 | 0.241 | 0.111 | 0.094 | 0.111 |
|  |  | 33.867 | | 4430-42-6 | Berteroin | 0.203 | 0.268 | 0.175 | 0.196 | 0.196 | 0.196 | 0.206 | 0.241 | 0.230 |
|  |  | 16.000 | | 1516-01-4 | 2,4-Hexadienenitrile | 3.891 | 4.351 | 2.894 | 3.594 | 3.585 | 3.415 | 3.646 | 3.936 | 4.029 |
|  |  | 7.267 | | 4786-20-3 | Crotononitrile (pract) | 0.058 | 0.076 | 0.045 | ND | ND | ND | 0.086 | 0.071 | 0.043 |
|  |  | 7.133 | | 18936-17-9 | 2-Methylbutyronitrile | 1.310 | 1.329 | 1.258 | 1.162 | 1.229 | 1.171 | 1.146 | 1.544 | 1.213 |
|  |  | 16.800 | | 3386-97-8 | Isothiocyanic acid, 3-butenyl ester | 49.153 | 52.311 | 53.930 | 54.897 | 55.584 | 55.810 | 50.461 | 53.909 | 54.195 |
|  |  | 11.800 | | 4786-24-7 | 3-Methylbut-2-enenitrile | 158.288 | 164.235 | 161.262 | 174.574 | 179.253 | 181.606 | 184.111 | 206.353 | 173.369 |
|  |  | 27.600 | | 206761-72-0 | Hexane, 1-isothiocyanato-3-methyl- | 1.172 | 0.977 | 0.904 | 1.302 | 1.271 | 0.851 | 1.206 | 1.244 | ND |
|  |  | 16.933 | | 40159-07-7 | 1,3-Dioxolane-2-propanenitrile, 2-methyl- | 0.504 | ND | 0.423 | 1.201 | ND | ND | 0.561 | 0.394 | 0.489 |
|  |  | 31.060 | | 2046-18-6 | 4-Phenylbutyronitrile | 0.770 | 0.686 | 0.786 | 0.330 | ND | ND | ND | 0.346 | 0.327 |
|  |  | 13.867 | | 5048-19-1 | 5-Hexenenitrile | 151.104 | 161.642 | 164.081 | 158.383 | 173.743 | 176.009 | 159.326 | 167.847 | 167.611 |
|  |  | 24.333 | | 59121-25-4 | 5-(methylsulfanyl) pentanenitrile | 16.266 | 15.142 | 18.229 | 21.091 | 21.641 | 18.478 | 15.079 | 22.673 | 15.856 |
|  |  | 24.933 | | 10412-98-3 | 5-Ketohexanenitrile | 0.878 | 0.880 | 0.562 | 0.889 | 1.174 | 0.732 | 1.073 | 1.365 | 1.533 |
|  |  | 17.467 | | 5048-25-9 | 6-HEPTENENITRILE | 1.625 | 1.388 | 1.361 | 1.116 | 1.107 | 0.922 | 0.789 | 0.924 | 0.755 |
|  |  | 29.467 | | 645-59-0 | 3-Phenylpropionitrile | 45.845 | 43.800 | 45.958 | 51.685 | 51.797 | 52.434 | 46.975 | 55.408 | 47.700 |
|  |  | 27.200 | | 140-29-4 | Phenylacetonitrile | 2.790 | ND | 2.720 | 2.690 | ND | 2.578 | 2.405 | ND | 2.440 |
|  |  | 11.800 | | 592-82-5 | Butyl isothiocyanate | 6.030 | 6.741 | 8.112 | 6.461 | 7.598 | 7.478 | 6.083 | 7.355 | 9.503 |
|  |  | 6.867 | | 624-92-0 | Dimethyl disulfide | 0.524 | 0.589 | 0.595 | 0.545 | 0.518 | 0.543 | 0.547 | 0.618 | 0.615 |
|  |  | 14.733 | | 3658-80-8 | Dimethyl trisulfide | 1.084 | 1.124 | 1.155 | 1.241 | 1.149 | 1.119 | 1.021 | 1.361 | 1.191 |
|  |  | 2.400 | | 75-15-0 | Carbon disulfide | 4.179 | 4.460 | 4.690 | 3.448 | 3.451 | 3.450 | 7.598 | 7.529 | 6.596 |
|  |  | 27.193 | | 529-19-1 | 2-Methylbenzonitrile | 2.598 | 2.655 | ND | ND | 2.595 | ND | 2.632 | 2.661 | ND |
|  |  | 31.800 | | 1885-38-7 | Cinnamonitrile | 0.327 | 0.289 | ND | 0.421 | 0.478 | 0.506 | 0.231 | 0.421 | 0.326 |
|  |  | 11.533 | | 1615-70-9 | 2,4-Pentadienenitrile | 14.100 | 15.125 | 14.111 | 14.113 | 14.412 | 14.238 | 13.319 | 16.936 | 16.097 |
|  |  | 26.533 | | 2179-59-1 | Allyl Propyl Disulfide | 0.341 | 0.400 | 0.468 | 0.363 | 0.378 | ND | 0.386 | ND | 0.524 |
|  |  | 33.067 | | 2257-09-2 | Phenethyl isothiocyanate | 0.843 | 0.821 | 0.906 | 1.264 | 1.253 | 1.153 | 0.868 | 1.041 | 1.022 |
|  |  | 18.933 | | 33522-03-1 | Cyclopentyl isothiocyanate | 29.463 | ND | 31.841 | 32.550 | 32.503 | ND | 31.354 | 30.185 | ND |
|  |  | 20.593 | | 4404-45-9 | Hexyl Isothiocyanate | 0.264 | 0.332 | ND | ND | ND | 0.329 | 0.352 | 0.298 | ND |
|  |  | 15.800 | | 629-08-3 | Heptanenitrile | 2.506 | 2.799 | 2.740 | 2.438 | 2.930 | 2.290 | 2.374 | 2.688 | 2.582 |
|  | | | **Total** | | | 500.858 | 486.564 | 523.639 | 536.652 | 558.642 | 526.030 | 540.337 | 593.910 | 514.022 |
| Heterocyclic compounds | Pyrazine | 18.260 | | 71257-37-9 | Pyrrolo[1,2-a]pyrazine,3,4-dihydro- | 0.818 | 0.800 | ND | ND | 0.649 | 0.796 | ND | 0.801 | 0.801 |
|  |  | 16.200 | | 14667-55-1 | Trimethyl-pyrazine | 7.616 | 8.082 | 10.218 | 8.667 | 9.345 | 9.202 | 7.841 | 9.924 | 9.178 |
|  |  | 22.200 | | 18433-98-2 | isopentyldimethylpyrazine,2-isopentyl-3,6-dimethylpyrazine | 0.122 | 0.080 | 0.134 | 0.090 | 0.075 | 0.087 | 0.079 | 0.103 | 0.096 |
|  |  | 22.860 | | 55138-73-3 | Pyrazine, 2,5-dimethyl-3-(1Z)-1-propen-1-yl- | 0.308 | ND | ND | ND | 0.383 | 0.215 | 0.062 | 0.063 | ND |
|  |  | 13.660 | | 123-32-0 | 2,5-Dimethylpyrazine | 6.553 | 6.093 | 5.633 | 0.693 | 0.861 | ND | ND | 1.737 | 1.737 |
|  |  | 15.733 | | 13360-64-0 | 2-Ethyl-5-methylpyrazine | 6.004 | 6.144 | 5.566 | 5.676 | 5.714 | 5.490 | 5.043 | 6.522 | 5.913 |
|  |  | 31.400 | | 13925-03-6 | 2-ethyl-6-methylpyrazine | 0.272 | 0.271 | 0.289 | ND | ND | 0.336 | 0.293 | 0.312 | 0.428 |
|  |  | 22.467 | | 23787-80-6 | 2-Acetyl-3-methylpyrazine | 5.580 | 5.289 | 5.329 | 5.000 | 4.911 | 4.604 | 4.918 | 5.105 | 5.456 |
|  |  | 18.533 | | 18138-05-1 | Pyrazine, 3,5-diethyl-2-methyl- (8CI,9CI) | 2.246 | 1.938 | 2.039 | 2.687 | 1.389 | 1.728 | 1.976 | 1.653 | 1.532 |
|  |  | 17.200 | | 13360-65-1 | 3-ethyl-2,5-dimethylpyrazine | 16.039 | 15.778 | 14.117 | 14.000 | 13.497 | 12.792 | 12.974 | 14.731 | 14.633 |
|  |  | 21.333 | | 23747-48-0 | 6,7-Dihydro-5-methyl-5(H)-cyclopentapyrazine | 0.532 | 0.684 | 0.605 | ND | ND | 0.437 | 0.469 | 0.500 | ND |
|  |  | 19.460 | | 55138-63-1 | Pyrazine, 2-methyl-5-(2-propen-1-yl)- | 1.766 | 2.408 | 1.779 | 1.431 | 1.373 | 1.566 | 2.463 | 1.495 | 1.541 |
|  | Furan | 37.333 | | 32780-06-6 | (S)-5-Hydroxymethyldihydrofuran-2-one | 0.928 | 1.011 | 1.143 | 0.886 | 0.704 | 0.694 | 0.901 | 0.873 | 1.075 |
|  |  | 23.667 | | 497-23-4 | 2(5H)-Furanone | 3.246 | 3.059 | 3.625 | 2.410 | 2.385 | 1.895 | 2.030 | 2.664 | 1.856 |
|  |  | 18.133 | | 1192-62-7 | 2-Acetylfuran | 5.710 | 5.661 | 5.730 | 5.123 | 5.850 | 4.598 | 4.609 | 5.742 | 6.366 |
|  |  | 25.933 | | 3658-77-3 | Furaneol | 3.871 | 4.903 | 2.839 | 2.162 | 2.167 | 2.191 | 1.986 | 2.257 | 1.601 |
|  |  | 22.133 | | 591-11-7 | 5-Methylfuran-2(5H)-one | 0.724 | 0.507 | 0.459 | 0.263 | 0.293 | ND | 0.298 | 0.355 | 0.403 |
|  |  | 21.867 | | 19340-56-8 | 3-butyldihydrofuran-2(3H)-one | 0.399 | ND | ND | 0.734 | ND | ND | 0.247 | 0.289 | 0.304 |
|  |  | 23.133 | | 3857-25-8 | 5-Methyl-2-furanmethanol | 0.236 | 0.193 | ND | 0.083 | 0.084 | ND | 0.122 | 0.176 | ND |
|  |  | 32.660 | | 10551-58-3 | (5-Formyl-2-furyl)methyl acetate | 1.421 | 0.730 | 1.075 | 0.951 | 1.224 | 0.661 | 0.603 | 0.547 | 0.675 |
|  | Pyridine | 9.727 | | 110-86-1 | Pyridine | 0.085 | ND | 0.139 | ND | ND | ND | ND | 0.119 | 0.107 |
|  |  | 36.067 | | 1452-77-3 | 2-Picolinamide | 0.189 | 0.165 | ND | 0.229 | 0.217 | ND | 0.157 | 0.123 | 0.140 |
|  |  | 36.600 | | 274-40-8 | Indolizine | 0.728 | 0.569 | 0.613 | ND | 0.803 | 0.848 | 0.543 | 0.698 | 0.644 |
|  | Pyran | 26.667 | | 39682-47-8 | 1.6-Anhydro-2.3-dideoxy-β-D-threo-hexopyranose | 0.732 | ND | 0.849 | 0.693 | 0.642 | 0.589 | 0.619 | 0.711 | ND |
|  |  | 33.667 | | 28564-83-2 | 2,3-Dihydro-3,5-dihydroxy-6-methyl-4(H)-pyran-4-one | 19.103 | 17.258 | 18.529 | 15.815 | 15.461 | 13.336 | 13.526 | 15.249 | 15.023 |
|  |  | 34.193 | | 1073-96-7 | 4H-Pyran-4-one, 3,5-dihydroxy-2-methyl- | ND | 1.071 | 1.481 | 1.752 | 1.641 | 1.380 | 0.593 | 0.394 | 0.710 |
|  | Pyrrole | 29.733 | | 616-45-5 | 2-Pyrrolidinone | 7.615 | 7.347 | 7.915 | 6.142 | 5.776 | 4.979 | 8.610 | 8.316 | 9.356 |
|  |  | 30.800 | | 1192-58-1 | N-Methylpyrrole-2-carboxaldehyde | 5.085 | 5.323 | 5.661 | 5.882 | 5.496 | 5.665 | 5.014 | 5.492 | 5.360 |
|  |  | 29.133 | | 108-27-0 | 5-Methyl-2-pyrrolidone | 1.981 | 1.412 | 1.422 | 0.492 | 0.502 | 0.494 | 2.048 | 2.117 | 1.538 |
|  | Thiazole | 23.860 | | 2289-75-0 | 4,5-Dimethylthiazol-2-amine | 0.937 | ND | 0.707 | 0.622 | 0.737 | 0.572 | 0.539 | 0.626 | ND |
|  |  | 26.000 | | 31883-01-9 | 5-ethyl-4-methylthiazole | 5.385 | 4.304 | 4.745 | 2.839 | 3.699 | 3.825 | 3.755 | 3.322 | 4.844 |
|  | Other heterocyclic compounds | 28.267 | | 77164-93-3 | (6-Methylpyrazin-2-yl)Methanol | ND | 2.239 | 2.521 | ND | ND | ND | 1.474 | 1.262 | ND |
|  |  | 22.200 | | 1003-31-2 | 2-Thiophenecarbonitrile | 0.426 | 0.413 | ND | 0.430 | 0.433 | ND | 0.517 | 0.584 | ND |
|  |  | 27.533 | | 636-72-6 | 2-Thiophenemethanol | 0.994 | 1.179 | 0.917 | 0.854 | 1.026 | 0.778 | 0.812 | ND | 0.903 |
|  |  | 22.400 | | 98-03-3 | 2-Thenaldehyde | 1.696 | 1.716 | 1.771 | 1.650 | 1.671 | 1.493 | 1.294 | 1.426 | ND |
|  |  | 19.400 | | 1003-04-9 | Tetrahydrothiophen-3-one | ND | 0.268 | ND | ND | 0.225 | 0.273 | 0.316 | ND | 0.271 |
|  | | | **Total** | | | 109.345 | 106.894 | 107.847 | 88.255 | 89.234 | 81.521 | 86.734 | 96.286 | 92.492 |
| Others | | 44.127 | | 102-93-2 | 3-Phenyl-propionamide | ND | 0.022 | 0.028 | 0.033 | 0.030 | 0.032 | ND | 0.033 | 0.029 |
|  |  | 23.667 | | 631-66-3 | Propanamide, 2-oxo- | 0.553 | 0.596 | 0.599 | 0.487 | 0.538 | 0.503 | 0.656 | 0.751 | 0.655 |
|  |  | 14.800 | | 2516-34-9 | Cyclobutylamine | 1.213 | 1.619 | 2.025 | ND | 2.306 | 2.740 | 1.837 | 1.468 | 2.202 |
|  |  | 30.000 | | 112-84-5 | Erucamide | 33.877 | 31.825 | 35.090 | 36.893 | 35.379 | 34.637 | 35.202 | 39.238 | 35.708 |
|  |  | 32.067 | | 628-02-4 | Hexanamide | 18.929 | 18.724 | 20.835 | 15.853 | 21.069 | 20.468 | 21.689 | 23.150 | 21.717 |
|  | | | **Total** | | | 54.573 | 52.786 | 58.577 | 53.266 | 59.322 | 58.379 | 59.385 | 64.641 | 60.312 |

**Note:** The content of volatile components in the table was calculated by the internal standard method using 2-octanol as the internal standard. Crude rapeseed oil (CRO), silica-degummed oil (SiRO), and APTES-SiO_2_-degummed oil (ARO).
